# A brown fat-enriched adipokine Adissp controls adipose thermogenesis and glucose homeostasis

**DOI:** 10.1038/s41467-022-35335-w

**Published:** 2022-12-10

**Authors:** Qingbo Chen, Lei Huang, Dongning Pan, Kai Hu, Rui Li, Randall H. Friedline, Jason K. Kim, Lihua Julie Zhu, David A. Guertin, Yong-Xu Wang

**Affiliations:** 1 Department of Molecular, Cell and Cancer Biology, University of Massachusetts Chan Medical School, Worcester, MA USA; 2 Program in Molecular Medicine, University of Massachusetts Chan Medical School, Worcester, MA USA; 3 Present Address: Key Laboratory of Metabolism and Molecular Medicine, Department of Biochemistry and Molecular Biology, Fudan University Shanghai Medical College, Shanghai, China

**Keywords:** Fat metabolism, Extracellular signalling molecules, Endocrine system and metabolic diseases

## Abstract

The signaling mechanisms underlying adipose thermogenesis have not been fully elucidated. Particularly, the involvement of adipokines that are selectively expressed in brown adipose tissue (BAT) and beige adipocytes remains to be investigated. Here we show that a previously uncharacterized adipokine (UPF0687 protein / human C20orf27 homolog) we named as Adissp (Adipose-secreted signaling protein) is a key regulator for white adipose tissue (WAT) thermogenesis and glucose homeostasis. Adissp expression is adipose-specific and highly BAT-enriched, and its secretion is stimulated by β3-adrenergic activation. Gain-of-functional studies collectively showed that secreted Adissp promotes WAT thermogenesis, improves glucose homeostasis, and protects against obesity. Adipose-specific *Adissp* knockout mice are defective in WAT browning, and are susceptible to high fat diet-induced obesity and hyperglycemia. Mechanistically, Adissp binds to a putative receptor on adipocyte surface and activates protein kinase A independently of β-adrenergic signaling. These results establish BAT-enriched Adissp as a major upstream signaling component in thermogenesis and offer a potential avenue for the treatment of obesity and diabetes.

## Abstract (web summary)

The signaling mechanisms regulating adipose thermogenesis have not been fully elucidated. Here, the authors show that a brown fat-enriched adipokine ADISSP promotes adipose thermogenesis and improves metabolic health independently of b-adrenergic receptor.

## Introduction

WAT browning and non-shivering thermogenesis are principally controlled by β-adrenergic receptor (β-AR) signaling^1,2^. Studies in mice have demonstrated that BAT and beige adipocytes can not only protect against obesity but also improve glucose homeostasis independent of changes of body weight^3–8^, although the underlying mechanisms of the latter effect remain unclear^8^. While agonists of β3-adrenergic receptor (β3-AR) have exhibited efficacy in energy expenditure and glucose uptake in human studies, their undesirable cardiovascular effects prevent their clinical use^9,10^. Therefore, there is an urgent need to identify thermogenic activators or signaling pathways that act independently of β-AR signaling.

While adipokines secreted from WAT have been extensively investigated, the secretomes of BAT and beige adipocytes have not been well characterized, let alone functionally studied. Although a few brown adipokines (batokines) have been identified, they are highly expressed in other tissues and/or have a very low level in BAT (and WAT)^11,12^. Moreover, their physiological functions attributable to adipose tissue secretion remain to be explored^12^. Thus, the secretory roles that are unique to BAT and beige adipocytes in energy homeostasis are largely unknown.

In this work, we identify a previously uncharacterized adipokine that is selectively expressed in adipose tissue and highly BAT-enriched. This adipokine acts as a key regulator in adipose thermogenesis and metabolic homeostasis at least in part through activating protein kinase A (PKA) independently of β-adrenergic signaling. Our study provides new molecular insights into our understanding of signaling mechanisms underlying adipose thermogenesis and the functions of BAT- and beige fat-enriched adipokines.

## Results

### Identification of a BAT-enriched adipokine

**Fig. 1:**
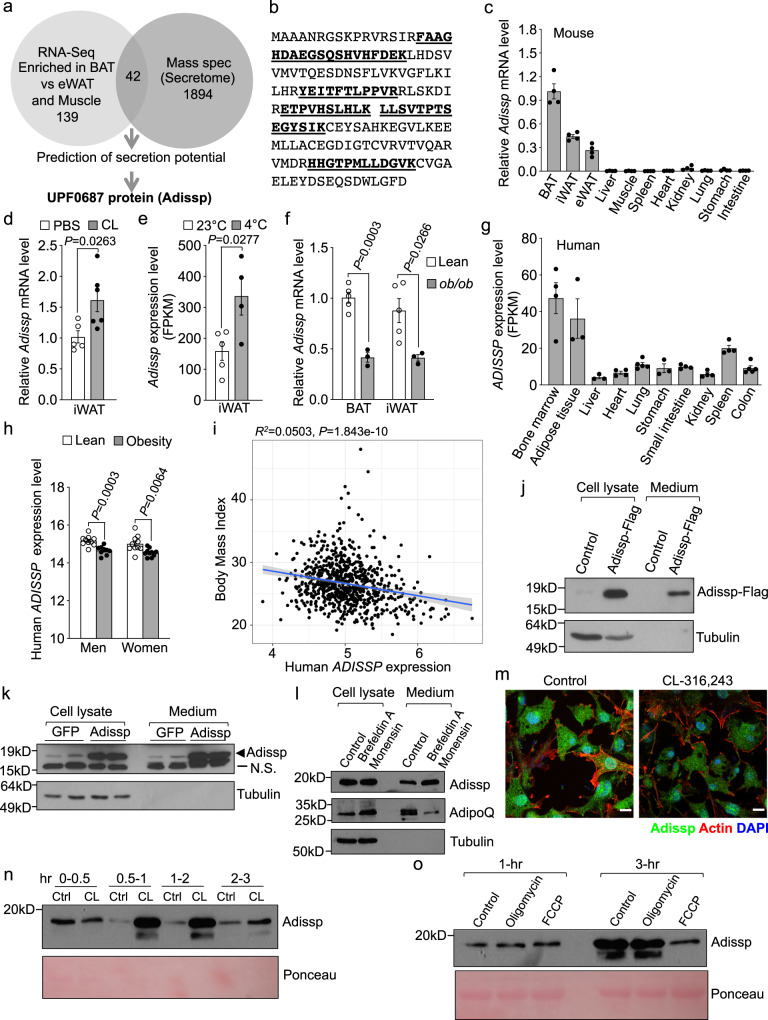
Identification of Adipose-secreted signaling protein (Adissp). **a** Strategies to identify brown adipocyte-secreted proteins. **b** Adissp-matched peptides identified by mass spectrometry are bold and underlined. **c** *Adissp* expression in wild-type (WT) mice tissues (*n* = 4). **d** *Adissp* expression in inguinal white adipose tissue (iWAT) from 2-month-old WT female mice administrated with PBS (*n* = 5) or CL-316,243 (CL) (*n* = 6). **e** *Adissp* expression in iWAT from WT mice housed at 22 °C (*n* = 5) or cold (4 °C) (*n* = 4) for 3 days from published data^18^. **f** *Adissp* mRNA expression in BAT and iWAT from 3-month-old male *ob/ob* mice (*n* = 3) and lean controls (*n* = 5). **g** Human *ADISSP* expression in different tissues (Bone marrow, Heart, Small Intestine, Kidney and Spleen, *n* = 4; Adipose tissue, Liver and Stomach, *n* = 3; Lung and Colon, *n* = 5) from published data^19^. **h** Expression of human *ADISSP* in subcutaneous adipocytes from lean (*n* = 10) and subjects with obesity (Men, *n* = 9; Women, *n* = 10) from published data^20^. **i** Linear regression analysis of Body Mass Index (BMI) and *ADISSP* expression from adipose tissue of 770 men from published data^21^. **j** Detection of secreted Adissp-Flag from brown adipocytes containing a Flag knock-in. **k** Detection of Adissp secretion from brown adipocytes. N.S., non-specific band. **l**, Detection of Adissp secretion from brown adipocytes treated with Brefeldin A and Monensin for 6 h. **m** Subcellular localization of Adissp in brown adipocytes treated with or without CL-316,243 for 45 min. Scale bar, 20μm. **n** Detection of Adissp secretion from brown adipocytes treated with CL-316,243 at indicated time intervals. **o** Detection of Adissp secretion from brown adipocytes treated with oligomycin or Carbonyl cyanide-4 (trifluoromethoxy) phenylhydrazone (FCCP) for 1 h or 3 h. In Fig. 1j–o, experiments were repeated twice independently with similar results. Data are mean ± s.e.m. *P* values were determined by two-tailed Student’s *t* test (**d**, **e**, **f** and **h**) or Linear regression analysis (**i**). Source data are provided as a Source Data file.

We aimed to identify BAT- and beige-selective adipokines that can act on adipose tissue to induce WAT browning. After removal of small molecules, serum-free conditioned medium collected from differentiated brown adipocyte culture was able to stimulate the expression of BAT-selective genes including *Ucp1* in primary inguinal adipocytes (Supplementary Fig. 1a), whereas heat-inactivated medium had little effect, suggesting that brown adipocytes secrete a protein(s) with browning activity. We exploited three independent strategies to identify this potential secreted protein (Fig. 1a). We first screened our previously published RNA-Seq datasets^13^ and identified 139 genes that are both abundantly and selectively expressed in BAT relative to epididymal WAT and skeletal muscle. We then performed proteomic analysis of serum-free conditioned medium collected from differentiated brown adipocytes and identified 1894 proteins. Among them, 1751 protein were also identified in conditioned medium of iWAT adipocytes^14^. Combining our proteomics data with the RNA-seq data led to 42 candidates (Supplementary Table 1). Interestingly, many of them are mitochondrial proteins. We envisioned that these mitochondrial proteins are likely secreted through extracellular vesicles, as mitochondrial components constitute a major part of extracellular vesicles^15^ and brown fat-derived exosomes^16^. Lastly, we used bioinformatics to predict whether any of the 42 candidates are potentially secreted proteins, and identified two proteins, Apolipoprotein C-I (Apoc1) and UPF0687. We focused on UPF0687, which is encoded by *1700037H04Rik*. *1700037H04Rik* and its human orthologue *C20orf27* are previously uncharacterized genes, and encode 19 kilodalton (kDa) products (Supplementary Fig. 1b) that are predicted by SecretomeP^17^ to be non-classically secreted proteins. Five peptides of this gene product were identified in our proteomic analysis (Fig. 1b). We named this adipokine as Adissp (Adipose-secreted signaling protein).

Quantitative PCR (qPCR) analysis showed that *Adissp* expression was restricted to adipose tissue and highly enriched in BAT, and was primarily present in mature adipocytes (Fig. 1c and Supplementary Fig. 1c, d). Daily β3-AR agonist CL-316,243 administration for 2 days and cold challenging at 4 °C for 3 days led to increased expression of *Adissp* in inguinal WAT (Fig. 1d, e)^18^, while acute treatment of adipocyte culture with CL-316,243 in vitro or acute cold exposure in vivo had no effect (Supplementary Fig. 1e, f). *Adissp* expression was decreased in BAT and inguinal WAT of *ob/ob* mice (Fig. 1f). We also analyzed publicly available gene expression datasets, and found that *ADISSP* expression was enriched in human adipose tissue (Fig. 1g)^19^, and was lower in adipocytes of human subjects with obesity compared with those of lean subjects (Fig. 1h)^20^. Importantly, we performed linear regression analyses on microarray data from subcutaneous adipose tissue samples of a cohort of 770 men and found that levels of *ADISSP* expression were negatively correlated with body mass index (BMI) (Fig. 1i)^21^.

To further confirm Adissp is a secreted protein, we used CRISPR/Cas9 system to knock in a Flag tag at endogenous *Adissp* locus immediately before its stop codon in immortalized brown preadipocytes. When probed with a Flag antibody, Adissp-Flag was detected in conditioned medium of differentiated knock-in adipocytes (Fig. 1j). We then obtained a commercial Adissp antibody that was generated against human ADISSP. Western blot analysis of BAT extracts from *Adissp* knockout mice validated the antibody (Supplementary Fig. 1g). Despite that the antibody had a relatively low reactivity to mouse Adissp protein (Supplementary Fig. 1h), we were able to detect endogenous Adissp present in conditioned medium of mouse brown adipocyte culture, and its level was increased when brown adipocytes were infected with adenovirus expressing *Adissp* (Fig. 1k). Based on equivalent loading of cell numbers, we estimated that at least 50% of produced Adissp in adipocyte culture was secreted into medium during a 16-h incubation. Similarly, in a competitive ELISA assay we developed (Supplementary Fig. 1i), we found that about 10% of total Adissp protein was secreted during a 3-h incubation. Moreover, ADISSP was shortlisted as a secreted protein in proteomic analyses of human adipocyte secretome^22^. Together, these results confirmed our proteomic data that endogenous Adissp is an adipokine.

Brefeldin A and monensin together block classical protein secretory pathway from the ER to the trans-Golgi apparatus cisternae. While adiponectin secretion was inhibited by brefeldin A and monensin as expected, Adissp secretion was instead increased (Fig. 1l). It has been reported that brefeldin A and monensin enhance the secretion of non-classically secreted proteins IL-1β and migration inhibitory factor^23,24^. These data provide additional evidence that Adissp is secreted via a non-classical secretory pathway.

We found that Adissp was present with a punctate or vesicular pattern (Fig. 1m). Importantly, treatment of adipocytes with β3-AR agonist CL-316,243 decreased Adissp staining (Fig. 1m), raising the possibility of increased secretion. To investigate this further, we collected conditioned medium at different time intervals. We found that Adissp secretion was stimulated by CL-316,243 during the first 2-h treatment, and this stimulation waned afterwards (Fig. 1n). Thus, β3-AR activation causes a spike of Adissp secretion.

Interestingly, Carbonyl cyanide-4 (trifluoromethoxy) phenylhydrazone (FCCP), an uncoupler of mitochondrial oxidative phosphorylation, inhibited Adissp secretion, whereas oligomycin, an inhibitor of ATP synthase, had no effect (Fig. 1o), which suggests that the uncoupling process, not ATP level per se, feeds back to negatively regulate Adissp secretion. The dynamic regulation of Adissp secretion by β3-AR activation and FCCP supports a potential role of this adipokine in adipose thermogenesis.

### Transgenic expression of *Adissp* enhances thermogenesis, improves glucose homeostasis, and protects against diet-induced obesity

**Fig. 2:**
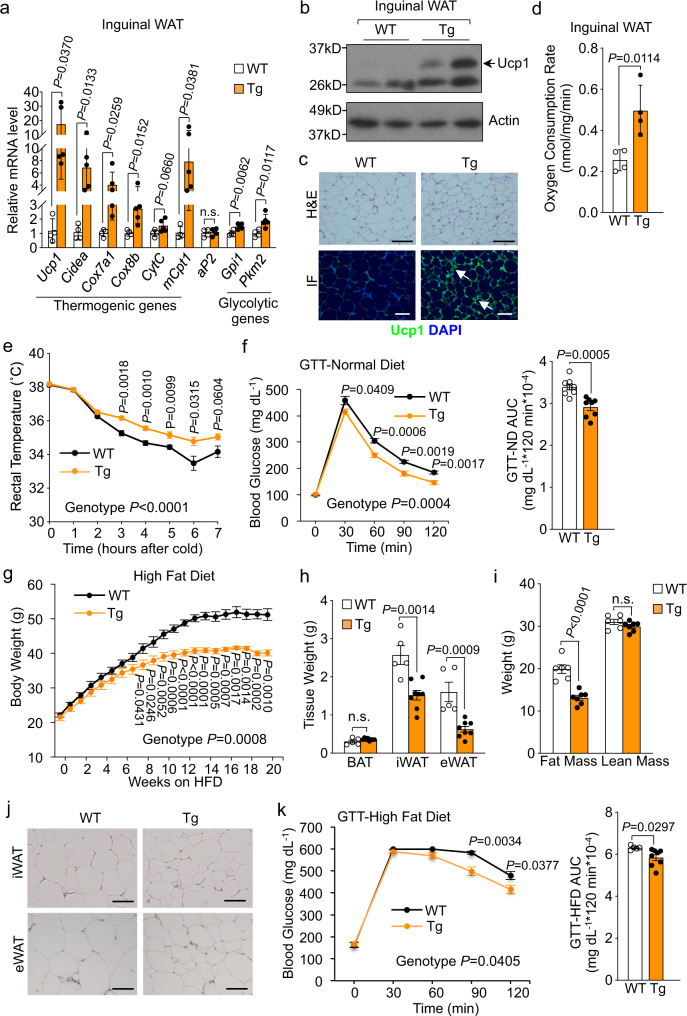
Targeted expression of *Adissp* enhances thermogenesis. **a** Gene expression in iWAT from 2-month-old male *Adissp* transgenic (Tg) mice (*n* = 5) and littermate controls (*n* = 4). **b** Western blot of Ucp1 in iWAT from *Adissp* Tg mice and controls (*n* = 2 mice per group). **c** Representative images of hematoxylin and eosin (H&E) staining and Ucp1 immunofluorescence (IF) staining in iWAT from *Adissp* Tg mice (*n* = 3 mice per group). Scale bar, 200 μm. **d** Oxygen consumption rate in iWAT from another cohort of 2-month-old male *Adissp* Tg mice and littermate controls (*n* = 4 mice per group). **e** Rectal temperature of 4-month-old female *Adissp* Tg mice (*n* = 9) and littermate controls (*n* = 5) during cold exposure. **f** Glucose tolerant test (GTT) in 10-month-old male *Adissp* Tg mice and littermate controls on normal chow diet (*n* = 8 mice per group). **g** Body weight of male *Adissp* Tg mice (*n* = 8) and littermate controls (n = 5) on high-fat diet (HFD). **h** Fat mass of mice from (**g**) after 24 weeks of HFD feeding. **i** Fat mass and lean mass of a second cohort of mice after 16 weeks of HFD feeding (WT, *n* = 6; Tg, *n* = 7). **j** Representative images of H&E staining of iWAT and epididymal white adipose tissue (eWAT) from mice in (**g**) after 24 weeks of HFD feeding. Scale bar, 200 μm. **k** GTT in mice from (**g**) on 16 weeks of HFD. Data are mean ± s.e.m. *P* values were determined by two-tailed Student’s *t* test (**a**, **d**, **h**, **i** and AUC in **f** and **k**) and two-way repeated measures ANOVA with post hoc test by Fisher’s LSD test (**e**, **f**, **g**, and **k**), n.s. (not significant). Source data are provided as a Source Data file.

Acute overexpression of *Adissp* with adenovirus in primary inguinal adipocytes increased expression of BAT markers *Ucp1*, *Cidea* and genes involved in mitochondrial oxidative metabolism and glycolysis (Supplementary Fig. 2a). General adipogenesis was not affected (Supplementary Fig. 2a, b). Co-treatment with CL-316,243 had an additive effect on *Ucp1* level (Supplementary Fig. 2c). Moreover, conditioned medium from HEK293 cells overexpressing *Adissp* induced *Ucp1* expression in primary inguinal adipocytes (Supplementary Fig. 2d). To determine whether Adissp is capable of driving WAT browning in vivo, we generated *aP2-Adissp* transgenic mice that selectively overexpressed *Adissp* in adipose tissue (Supplementary Fig. 3a). Serum collected from *Adissp* transgenic mice, which had a higher Adissp level in circulation (Supplementary Fig. 3b), increased thermogenic genes expression in inguinal adipocyte culture (Supplementary Fig. 3c). On a normal chow diet, while there was no difference in body weight and food intake (Supplementary Fig. 3d, e), the transgenic mice had elevated expression of thermogenic and glycolytic genes in inguinal WAT, but not in BAT or epididymal WAT, compared with littermate controls (Fig. 2a, b and Supplementary Fig. 3f, g). Haemotoxylin and Eosin (H&E) staining and Ucp1 immunofluorescence staining confirmed inguinal WAT browning that possessed numerous small, UCP1-positive adipocytes (Fig. 2c and Supplementary Fig. 3h). Consistent with this, injection of *Adissp* adenovirus into the inguinal WAT also promoted its browning (Supplementary Fig. 3i, j). However, we noticed that iWAT browning induced by Adissp was not as extensive as what occurred in mice challenged by cold or treated with CL-316,243, or in Hlx transgenic mice^6^, in which the inguinal WAT is visibly brown. As expected, the transgenic mice displayed a significantly higher oxygen consumption rate in inguinal WAT (Fig. 2d), and their body temperature is higher compared with that of control mice at cold (Fig. 2e). These data revealed that Adissp promotes thermogenic activation and augments oxygen consumption. Interestingly, although tending to be higher in transgenic mice, whole-body energy expenditure was not significantly different (Supplementary Fig. 3k, l), which might be due to the stress condition in metabolic cages^25^, and/or the detection limit of indirect calorimetry^26^ along with variation of individual animals. Of note, others have also reported no difference in energy expenditure in animals with a browning phenotype at basal conditions^27–29^.

The *Adissp* transgenic mice were more glucose tolerant (Fig. 2f), consistent with the notion that WAT browning improves glucose homeostasis^3,5,6,30–32^. On a high-fat diet (HFD), the transgenic mice gained significantly less body weight and fat mass (Fig. 2g, h), despite similar food intake as control mice (Supplementary Fig. 4a). This was confirmed in a second cohort of HFD-fed mice (Fig. 2i and Supplementary Fig. 4b). Inguinal WAT and epididymal WAT adipocytes are substantially smaller (Fig. 2j). Moreover, the transgenic mice fed on HFD also displayed better glucose homeostasis (Fig. 2k). Together, inguinal WAT browning in the *Adissp* transgenic mice improves glucose homeostasis and protects against HFD-induced obesity.

### Adissp is required for inguinal WAT browning

**Fig. 3:**
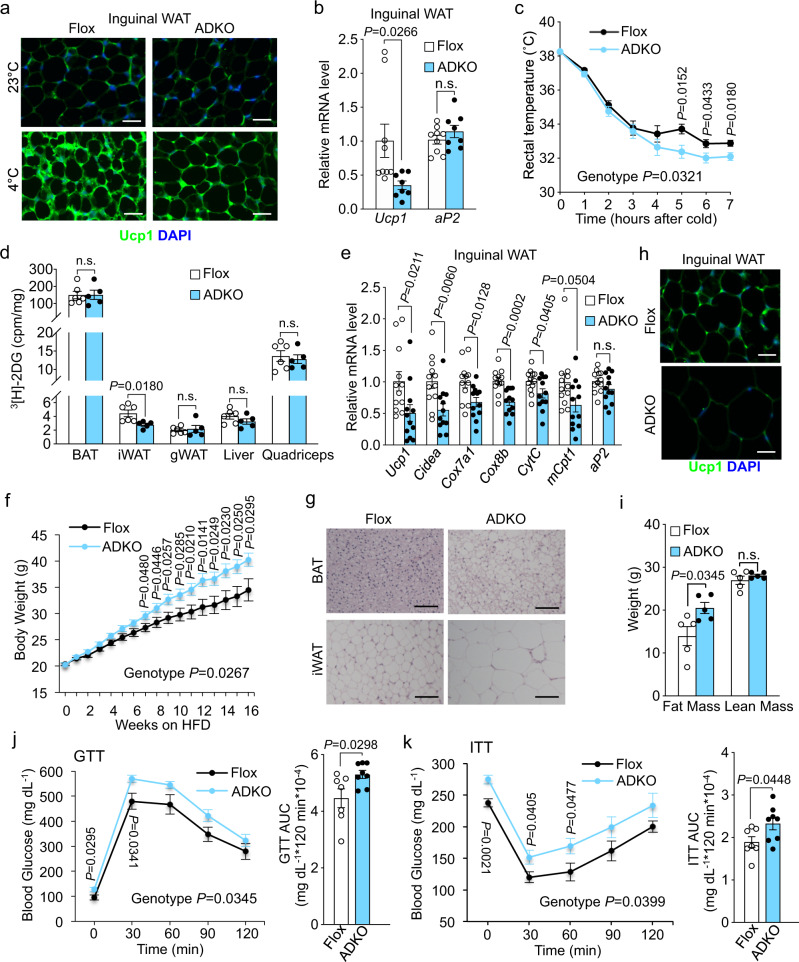
Adissp is required for iWAT browning and energy homeostasis. **a** Representative images of Ucp1 immunofluorescence staining in iWAT from 3-month-old male *Adissp* adipose-specific knockout (ADKO) mice and Flox controls housed at room temperature and after 8 h cold exposure (*n* = 3 mice per group). Scale bar, 200 μm. **b** Gene expression analysis in iWAT from 5-month-old female *Adissp* ADKO mice (n = 8) and Flox controls (*n* = 9) after 7 h cold exposure. **c** Rectal temperature of mice from (**b**) during cold exposure. **d** Quantification of ^3^H-2DG uptake in indicated tissues from female *Adissp* ADKO mice (*n* = 5) and Flox controls (n = 6) after 3 h cold exposure. **e** Gene expression analysis in iWAT from 2 days CL-316,243 (1 mg/kg body weight) administrated 3-month-old female mice (n = 12 mice per group). **f** Body weight of male *Adissp* ADKO mice (*n* = 15) and Flox controls (*n* = 12) on HFD. **g**, **h** Representative images of H&E staining in BAT and iWAT (**g**) and Ucp1 immunofluorescence staining in iWAT (**h**) from mice in (**f**) on 21 weeks of HFD (*n* = 3 mice per group). Scale bar, 200 μm. **i** Fat mass and lean mass of a second cohort of mice after 16 weeks of HFD feeding (n = 5 mice per group). **j**, GTT in mice from (**f**) on 18 weeks of HFD (Flox, *n* = 7; ADKO, n = 8). **k** Insulin tolerant test (ITT) in mice from (**f**) on 19 weeks of HFD (Flox, *n* = 7; ADKO, *n* = 8). Data are mean ± s.e.m. *P* values were determined by two-tailed Student’s *t* test (**b**, **d**, **e**, **i** and AUC in **j** and **k**) and two-way repeated measures ANOVA with post hoc test by Fisher’s LSD test (**c**, **f**, **j**, and **k**), n.s. (not significant). Source data are provided as a Source Data file.

To investigate the thermogenic function of endogenous Adissp, we generated *Adissp flox/flox* (Flox) mice, and then crossed the Flox mice with *Adiponectin-Cre* mice^33^ to generate *Adissp* adipose-specific knockout (ADKO) mice. Analysis of *Adissp* mRNA expression from multiple tissues confirmed that the deletion was specific to WAT and BAT (Supplementary Fig. 5a). At room temperature with a normal chow diet, the ADKO mice had similar body weight and food intake as littermate control mice, and no difference in thermogenic genes expression was observed in adipose tissues including inguinal WAT (Supplementary Fig. 5b-f). We also failed to observe any difference in energy expenditure and physical activity (Supplementary Fig. 5g, h). We then asked whether Adissp is required for beige fat biogenesis at thermogenic demanding conditions. As expected, we found that cold exposure stimulated the generation of UCP1-positive beige adipocytes in the inguinal WAT of control mice; however, beige fat formation was strikingly impaired in ADKO mice (Fig. 3a), which was further confirmed by analysis of *Ucp1* mRNA expression (Fig. 3b). Correspondingly, the body temperature was lower in the ADKO mice than control mice (Fig. 3c). Interestingly, ADKO mice contained more inguinal WAT mass after acute cold exposure (Supplementary Fig. 5i), reflecting decreased fat burning. Compromised inguinal WAT browning also led to decreased glucose uptake (Fig. 3d). We next determined whether Adissp is indispensable for β3-adrenergic signaling-induced inguinal WAT browning. Similar to what occurred at condition of cold challenging, administration of CL-316,243 led to robust inguinal WAT browning in control mice but not in ADKO mice (Fig. 3e and Supplementary Fig. 5j), and ADKO lost less inguinal WAT mass (Supplementary Fig. 5k). Together, these results suggest that endogenous Adissp is critically required for both cold- and β3-adrenergic agonist-stimulated beige fat formation and thermogenesis.

### The ADKO mice are prone to HFD-induced obesity and abnormal glucose metabolism

To examine the physiological requirement of Adissp for maintaining energy balance during energy overload, we challenged the ADKO mice with HFD. The ADKO mice consumed similar amounts food as control mice, yet gained significantly more body weight (Fig. 3f and Supplementary Fig. 6a), which was associated with substantially larger adipocytes with diminished UCP1 staining (Fig. 3g, h). EchoMRI measurement of a second cohort of ADKO mice confirmed that the body weight difference was due to fat mass, not lean mass (Fig. 3i and Supplementary Fig. 6b). We noted that the wild-type littermates of ADKO mice gained less body weight than wild-type littermates of *Adissp* transgenic mice when fed HFD for a similar time period (Fig. 2g and Fig. 3f); this is likely due to their differences in genetic background and food intake. The ADKO mice and littermate controls consumed 2.6 grams per day (Supplementary Fig. 6a), while the *Adissp* transgenic mice and littermate controls consumed about 3.2 grams per day (Supplementary Fig. 4a). Remarkably, both steady-state and fasting glucose levels were higher, and glucose tolerance and insulin sensitivity were deteriorated in the ADKO mice fed on HFD for 18 and 19 weeks (Fig. 3j, k). Glucose tolerance and insulin tolerance tests were also performed at week 5 and week 6 of HFD feeding, respectively, when there was no difference in body weight compared with littermate controls, and similar results were obtained (Supplementary Fig. 6c, d). Thus, impaired beige adipocyte development caused by *Adissp* ablation produced serious detrimental metabolic phenotypes that were opposite to those of *Adissp* transgenic mice.

### Adissp promotes thermogenesis and improves glucose homeostasis through both paracrine and endocrine signaling

BAT-secreted Adissp is unlikely to have significant contribution to WAT browning due to the substantially smaller mass of this depot. Indeed, neither BAT-selective overexpression nor BAT-selective deletion of *Adissp* had any effect on inguinal WAT browning (Supplementary Fig. 7a, b). Therefore, the observed phenotypes of inguinal WAT thermogenesis in *Adissp* transgenic mice and ADKO mice were likely due to paracrine signaling rather than endocrine signaling.

**Fig. 4:**
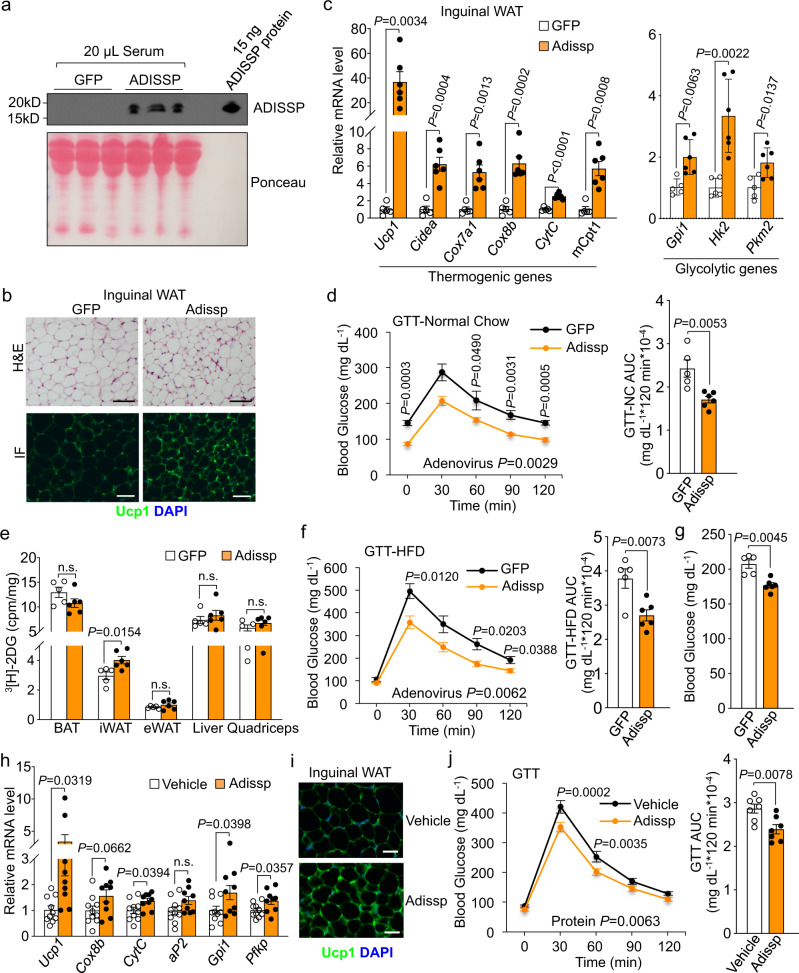
Endocrine action of Adissp. **a** Detection of circulating ADISSP protein (*n* = 3 mice per group). **b** Representative images of H&E staining and Ucp1 immunofluorescence staining in iWAT from indicated adenovirus-injected mice (*n* = 3 mice per group). Scale bar, 200 μm. **c** mRNA expression of indicated genes in iWAT from adenovirus-injected mice (GFP, *n* = 5; Adissp, *n* = 6). **d** GTT in adenovirus-injected mice fasted for 5 h (GFP, *n* = 5; Adissp, *n* = 6). **e** Quantification of ^3^H-2DG uptake in indicated tissues from adenovirus-injected mice (GFP, *n* = 5; Adissp, *n* = 6) after 5 h fasting. **f** GTT in HFD-induced obese mice that were fasted overnight (GFP, *n* = 5; Adissp, *n* = 6). **g** Basal glucose of mice in (**f**) that were fasted for 5 h (GFP, *n* = 5; Adissp, n = 6). **h** Gene expression in iWAT from 3-month-old WT male mice intravenously injected with Adissp protein or vehicle once a day for 9 days (Vehicle, n = 10; Adissp, *n* = 9). **i** Representative images of Ucp1 immunofluorescence staining in iWAT from mice in (**h)** (*n* = 7 mice per group). Scale bar, 200 μm. **j** GTT in another cohort of 3-month-old WT male mice at day 7 of injection (*n* = 7 mice per group). Data are mean ± s.e.m. *P* values were determined by two-tailed Student’s *t* test (**c**, **e**, **g**, **h** and AUC in **d**, **f**, and **j**) and two-way repeated measures ANOVA with post hoc test by Fisher’s LSD test (**d**, **f**, and **j**), n.s. (not significant). Source data are provided as a Source Data file.

We next determined whether Adissp is capable of functioning in an endocrine manner. We tail-vein-infused adenoviruses expressing either *GFP*, mouse *Adissp*, or human *ADISSP* into C57BL/6 J wild-type mice. As shown with human *ADISSP* adenovirus, this injection resulted in acute expression of ADISSP in the liver and its secretion into circulation with a concentration of about 0.45 μg/mL (Fig. 4a and Supplementary Fig. 8a). Importantly, no leaky expression of *ADISSP* was observed in the adipose tissue (Supplementary Fig. 8b). Circulating Adissp led to a robust browning of inguinal WAT, as evidenced by strong induction of *Ucp1*, *Cidea*, mitochondrial genes and glycolytic genes, and occurrence of numerous smaller, Ucp1-positive adipocytes (Fig. 4b, c and Supplementary Fig. 8c), whereas no effect was observed in BAT and epididymal WAT (Supplementary Fig. 8d, e). In addition, expression of key metabolic genes in the liver was not altered (Supplementary Fig. 8f). Importantly, inguinal WAT browning was associated with lower steady-state glucose level, higher glucose tolerance, and increased glucose uptake (Fig. 4d, e). To examine potential benefits of Adissp for glycemic control in an obese setting, we adenovirally expressed *Adissp* in the liver of HFD-induced obese mice. Similar to what observed in the lean mice, obese mice receiving *Adissp* adenovirus displayed a strong improvement of basal glucose level and glucose tolerance compared with obese mice receiving *GFP* adenovirus (Fig. 4f, g). Together, our data demonstrated that secreted Adissp from liver acts in an endocrine manner to promote inguinal WAT browning and markedly improve glucose homeostasis.

We purified recombinant His-tagged Adissp protein from HEK293 cells medium (Supplementary Fig. 8g). We then intravenously injected wild-type mice with Adissp protein daily for 9 days, which resulted in circulating level at 3.3 µg/mL. Compared with vehicle, Adissp protein induced thermogenic gene expression and inguinal WAT browning (Fig. 4h, i and Supplementary Fig. 8h). Adissp protein injected mice also exhibited improvement of glucose tolerance (Fig. 4j). We noted that recombinant Adissp protein appeared to be less effective than adenoviral infusion; this may be due to the lengthy purification process that might affect Adissp activity (Supplementary Fig. 8i). Our results provide additional evidence that pharmacological levels of Adissp can induce inguinal WAT browning through endocrine signaling.

### Secreted Adissp activates PKA signaling to exert its thermogenic function

**Fig. 5:**
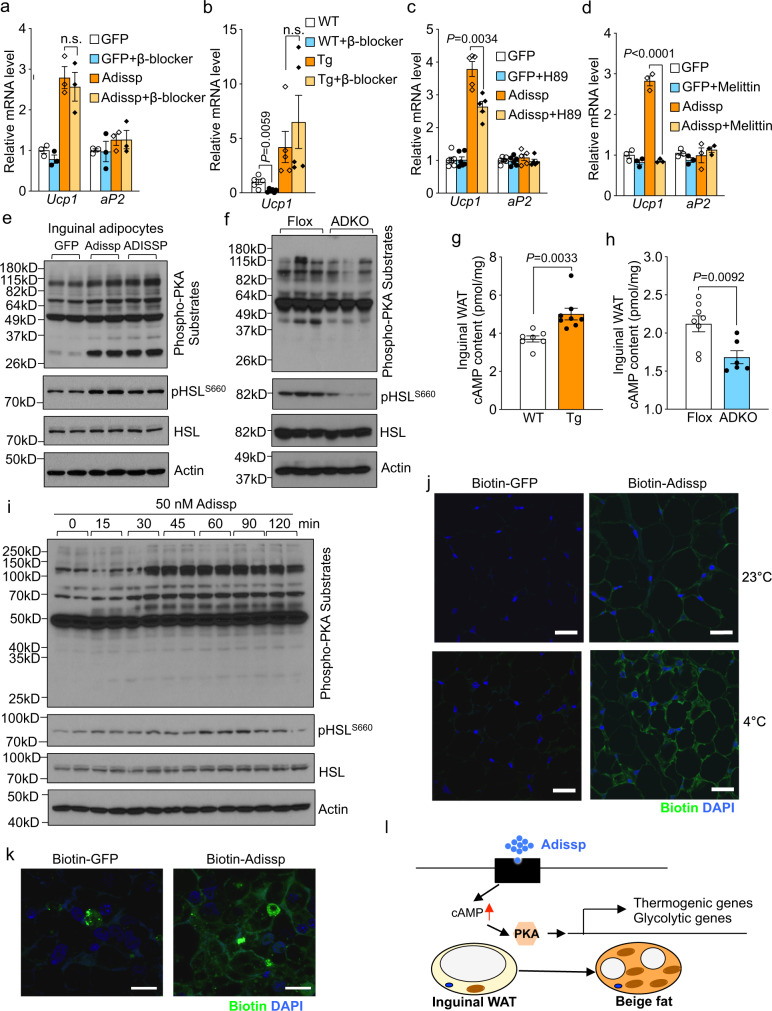
Adissp functions through PKA signaling. **a** Gene expression in inguinal adipocytes that overexpressing *Adissp* and treated with β-blocker (100 nM) for 24 h (*n* = 3). **b** *Ucp1* expression in iWAT from 2-month-old female *Adissp* Tg mice and WT controls after administrated with β-blocker (25 mg/kg body weight) for 7 days (WT, *n* = 5; WT + β-blocker, *n* = 6; Tg, *n* = 5; Tg+β-blocker, *n* = 5). **c**, **d** Gene expression in inguinal adipocytes overexpressing *Adissp* and treated with 30 μM H-89 dihydrochloride hydrate (H89) (GFP and GFP + H89, *n* = 6; Adissp and Adissp+H89, *n* = 5) for 2 h (**c**) or 1 μM Melittin (*n* = 3) for 24 h (**d**). **e** Western blot of phosphorylated protein kinase A (PKA) substrates and Hormone-sensitive lipase (HSL) in inguinal adipocytes transduced with indicated adenovirus. **f** Western blot of phosphorylated PKA substrates and HSL in iWAT from 3-month-old female *Adissp* ADKO mice and Flox controls after 3 h cold exposure. **g** cAMP levels in iWAT from 2-month-old male *Adissp* Tg mice (*n* = 8) and WT controls (*n* = 7). **h** cAMP levels in iWAT from mice in (**f**) (Flox, *n* = 8; ADKO, *n* = 6). **i** Western blot of phosphorylated PKA substrates and HSL in brown adipocytes treated with Adissp protein. **j** Binding of biotin-labeled Adissp to iWAT from WT mice housed at 23 °C or cold (4 °C) challenged for 8 h (*n* = 3 mice per group). Scale bar, 20 μm. **k** Binding of biotin-labeled Adissp to brown adipocyte surface. Scale bar, 20 μm. **l** Working model of Adissp. Data are mean ± s.e.m. *P* values were determined by two-tailed Student’s *t* test, n.s. (not significant). Source data are provided as a Source Data file.

We sought to determine the signaling pathway responsible for the thermogenic function of Adissp. β3-AR and downstream PKA activation play a pivotal role in adipose thermogenesis^1,2^. Our in vitro adipocyte culture data showing that Adissp can induce *Ucp1* expression at basal condition and has an additive effect in the presence of β3-AR agonist (Supplementary Fig. 2c) indicate that β3-AR is unlikely to be involved. Indeed, *Ucp1* expression induced by adenoviral expression of Adissp in adipocytes was not blocked by propranolol hydrochloride (β-blocker), a pan-β-receptor antagonist (Fig. 5a). To confirm this in vivo, *Adissp* transgenic and control mice were administered with β-blocker. As expected, β-blocker markedly abolished *Ucp1* expression in inguinal WAT of control mice, but had no effect on *Ucp1* expression in *Adissp* transgenic mice (Fig. 5b). Next, *Adissp* transgenic mice and ADKO mice were housed at 30 °C and fed a normal chow diet for 1 month and 2 months, respectively. Differences in adipocyte morphology and Ucp1 level were observed in both inguinal WAT and BAT, compared with their respective littermate controls (Supplementary Fig. 9a-d). Although similar body weights as those of their respective littermate controls, WAT mass was lower in the *Adissp* transgenic mice and was higher in the ADKO mice (Supplementary Fig. 9e, f). Thus, Adissp-regulated thermogenesis is still maintained at thermoneutral condition. These results together suggest that Adissp acts independently of β-AR. Interestingly, we found that induction of *Ucp1* expression by Adissp was reduced by a merely 2-h treatment with the PKA inhibitor H89 and was blocked by Melittin, an inhibitor for Gαs subunit of the heterotrimeric G protein (Fig. 5c, d), raising the idea that a G protein and PKA signaling axis might be responsible for the thermogenic activity of Adissp. We thus examined PKA activation by western blot analysis using an antibody that detects a bulk of phosphorylated PKA substrates and an antibody against PKA-phosphorylated hormone-sensitive lipase (pHSL). We found that phosphorylation of PKA substrates including HSL was increased in adipocyte culture infected with *Adissp* adenovirus (Fig. 5e) as well as in inguinal WAT of *Adissp* transgenic mice (Supplementary Fig. 10a), whereas inguinal WAT of ADKO mice had a decreased phosphorylation of PKA substrates and HSL (Fig. 5f). To provide independent evidence for PKA activation, we measured intracellular cAMP content. cAMP level was increased in primary inguinal adipocytes infected with *Adissp* adenovirus (Supplementary Fig. 10b) and in inguinal WAT depot of *Adissp* transgenic mice (Fig. 5g), and was decreased in inguinal WAT of ADKO mice (Fig. 5h). We next used recombinant Adissp protein to directly examine PKA activation. Phosphorylation of PKA substrates in adipocyte culture was increased by purified Adissp protein in a dose-dependent (Supplementary Fig. 10c) and time-dependent manner, with peak activation at 45-60 min (Fig. 5i). Moreover, phosphorylation of PKA substrates was induced in inguinal WAT of Adissp protein administered mice (Supplementary Fig. 10d). These results together demonstrate that secreted Adissp activates a G protein-cAMP-PKA signaling pathway.

Our data indicate that Adissp likely binds to a cell surface receptor distinct from β-AR to activate downstream signaling in adipocytes. To provide evidence for the existence of such a receptor, conditioned medium from HEK293 cells expressing Adissp fused to the C-terminus of secreted alkaline phosphatase (SEAP) was incubated with frozen tissue slices. Binding of SEAP-Adissp was determined by examining alkaline phosphatase activity. Whereas liver, skeletal muscle and brain had a near background binding signal, a specific binding of Adissp to adipose tissue sections was detected, which can be competed away by incubation with Adissp-containing conditioned medium (Supplementary Fig. 10e, f). In a second set of experiments, recombinant Adissp protein was labeled with biotin, incubated with inguinal WAT tissue sections, and stained with Alexa Fluor 488-conjugated Streptavidin. We found that Adissp, not GFP control, bound to adipose sections (Fig. 5j). Interestingly, the binding was increased in adipose tissue of mice challenged by cold (Fig. 5j). Adissp was also bound to cell surface of cultured adipocytes (Fig. 5k). Together, our data suggest that Adissp activates PKA signaling through binding to a putative receptor at adipocyte surface (Fig. 5l).

## Discussion

Our study shows that Adissp, encoded by a previously uncharacterized gene, is a bona fide, BAT-enriched adipokine. While a few batokines were previously identified, they are highly expressed in other tissues and/or have a very low expression in adipose tissue, and the importance of endogenous levels of these proteins secreted from adipose tissue was unknown^12^. Adissp is almost exclusively expressed in adipose tissue and, based on RNA-Seq data, *Adissp* mRNA is one of the most abundant transcripts in BAT. Adipose-specific *Adissp* deletion decreases cAMP content and PKA activity, suppresses inguinal WAT browning, and leads to HFD-induced obesity and hyperglycemia. Thus, Adissp is one of the few BAT-enriched adipokines known to date and plays an indispensible role in metabolic physiology^34^. Currently we were not able to measure endogenous, circulating Adissp level, which requires a more sensitive assay. In this regard, it is important to note that our data suggest that endogenous Adissp regulates inguinal WAT thermogenesis through paracrine signaling, and BAT-secreted Adissp has little contribution given the substantially smaller mass of this fat depot. Yet, when administered at a pharmacological level in circulation, Adissp can have a robust effect through endocrine action.

The mechanisms underlying the beneficial effect of WAT browning on glucose metabolism were unclear^8^. One important finding in our study is that Adissp markedly improves glucose homeostasis independent of body weight. Recent work by others has shown that mild cold promotes glycolytic beige fat formation when β-adrenergic signaling is blocked^35^. We found that inguinal WAT browning promoted by Adissp is also associated with increased expression of glycolytic genes, and interestingly, this increase can occur at room temperature and without blocking β-adrenergic signaling. The effect of Adissp on glucose metabolism could be in part attributed to glycolysis. Alternatively, Adissp may additionally have a signaling role in glycemic control. It has been postulated that beige fat secretes factors to directly regulate glucose metabolism^36,37^.

Adissp binds to a putative cell surface receptor and subsequently activates downstream PKA to promote WAT browning and metabolic healthy independently of β-AR activation. While the PKA signaling is also required in BAT, the effect of Adissp was only manifested under a thermoneutral condition, not at ambient temperature. At ambient temperature, BAT has a constitutively active, basal β3-AR signaling and hence high basal PKA activity due to its abundant sympathetic innervation, which likely renders Adissp less important. On the other hand, in the inguinal WAT, activation of both pathways, converging on downstream PKA activation, appears to have a synergistic effect, suggesting a concerted action. This is also consistent with our observations that acute β3-AR activation stimulates Adissp secretion and chronic β3-AR activation increases Adissp expression. Thus, Adissp serves to further ensure a full thermogenic response in inguinal WAT. In summary, our study provides novel molecular insights into our understating of signaling pathways underlying adipose thermogenesis and suggests a therapeutic potential of Adissp for the treatment of obesity and diabetes.

## Methods

### Source and maintenance of cell lines

HEK293 cell line used in this study was purchased from ATCC (American Type Culture Collection). Immortalized brown preadipocytes were generated previously^38^. Primary preadipocytes used in this study were isolated from inguinal WAT of experimental-housed mice. HEK293 cells and immortalized brown preadipocytes were maintained in DMEM medium (Gibco) containing 10% FBS, 1× Pen/Strep (Penicillin/Streptomycin) at 37 °C in 5% CO_2_. Primary preadipocytes were maintained in DMEM media with 20% FBS and 1× Pen/Strep before differentiation_._

### Adipocyte culture

Immortalized brown preadipocytes were generated previously^38^. Brown preadipocytes were immortalized by infection with the retrovirus expressing SV40 Large T antigen and selected with G418. At day −2 of differentiation, 70% confluent brown preadipocytes were cultured in DMEM medium (Gibco) containing 10% FBS (Atlanta Biologicals) and 1× Pen/Strep and supplemented with 20 nM insulin (Sigma) and 1 nM triiodotyronin (Sigma) (differentiation medium). At day 0, differentiation was induced by culturing cells in differentiation medium supplemented with 0.5 mM isobutylmethylxanthine (Acros organics), 0.5 μM dexamethasone (Acros organics), and 0.125 mM indomethacin (Alfa Aesar) for 2 days. Cells were then changed back to differentiation medium and fresh medium was replenished every 2 days. Inguinal stromal-vascular fractions (SVF) were isolated from 2-week-old C57BL/6 J mice with collagenase D (Roche) digestion and differentiated as described previously^39^. In brief, inguinal WAT was digested in isolation buffer (2.4 U/mL Dispase II (Roche, #04942078001), 100 mM HEPES (pH 7.4), 1.5 U/mL collagenase D (Roche, #11088882001) and 10 mM CaCl_2_) at 37 °C for 45 min. The samples were filtered through 70-μm cell strainers, and were than centrifuged at 700 g for 5 min at room temperature. The stromal-vascular cells were plated in 6-well or 12-well plates in DMEM media with 20% FBS and 1× Pen/Strep. Inguinal preadipocytes differentiation was started by culturing confluent cells in DMEM/F12 (Gibco) medium containing 10% FBS, 1× Pen/Strep, 850 nM insulin, 1 nM triiodotyronin, 0.5 mM isobutylmethylxanthine, 0.5 μM dexamethasone, and 0.125 mM indomethacin. After 2 days, cells were cultured in DMEM/F12 medium containing 10% FBS, 1× Pen/Strep, 850 nM insulin and 1 nM triiodotyronin and cell medium was changed every 2 days. For conditioned medium experiment, after removal of small molecules with a 10-kDa cut-off filter, serum-free conditioned medium collected from immortalized brown adipocyte culture was used to treat primary inguinal adipocytes. Heat-treated medium was heated at 95 °C for 10 min. Both brown and inguinal adipocyte were fully differentiated and harvested at day 6. In some experiments, differentiated adipocytes were treated with 10 μM CL-316,243 (R&D Systems, #1499), 100 nM propranolol hydrochloride (Sigma, #P0884), 30 μM H-89 dihydrochloride hydrate (Sigma, #B1427), or 1 μM Melittin (Tocris, #1193) as indicated.

### Identification of brown fat-enriched adipokines

Conditioned medium containing secreted proteins from immortalized brown adipocytes was concentrated with a 3-kDa cut-off centrifugal filter (Millipore). Proteomics was done by MS Bioworks (Ann Arbor, MI). Ten micrograms protein was subjected to SDS-PAGE with multiple band excision and in-gel digestion. The digested sample was analyzed by Liquid Chromatography with tandem mass spectrometry (LC-MS/MS). Protein identification was performed with Mascot. Protein visualization and validation was performed with Scaffold. The mass spectrometry proteomics data have been deposited to the ProteomeXchange Consortium via the PRIDE^40^ partner repository with the dataset identifier PXD038041 and 10.6019/PXD038041. We screened previously published RNA-seq dataset (GSE56367)^13^ and generated a list of genes abundantly expressed in BAT (FPKM > 40) that are >3-fold and >5-fold relative to epididymal WAT and skeletal muscle, respectively. Overlapping genes from the above analyses were then predicted for secretion potential using SignalP and SecretomeP (www.cbs.dtu.dk/services)^17,41^ and the Universal Protein Resource (UniProt).

### Generation of *Adissp*-*flag* knockin cells

CRISPR/Cas9 system was used to knock in a Flag tag immediately before the stop codon at the endogenous *Adissp* locus in immortalized brown preadipocytes. Oligonucleotides (F: CACCGCTCGGCTTCGACTGAGGCC, R: AAACGGCCTCAGTCGAAGCCGAGC) were cloned into pX330-puro vector^42^ for expression of the guide RNA. A 1-kb flanking *Adissp* genomic fragment with the Flag tag inserted before the stop codon was produced by PCR and cloned into pBluescript II vector. The above plasmids were co-transfected into brown preadipocytes and selected with puromycin (Sigma). Cells were re-plated without puromycin and single cell clones were examined by genotyping. Correctly targeted single cell clones were further verified by sequencing of produced *Adissp* transcript.

### Animals

C57BL/6J wild-type mice (Stock No. 000664) were obtained from the Jackson Laboratory. 3-month-old male C57BL/6J mice were used for adenoviruses or protein injection. *Ucp1* knockout mice were obtained from David A. Guertin lab. *Adissp* transgenic mice were generated by core facility of UMASS Medical School. *Adissp* cDNA was fused downstream of the 5.4 kb *aP2* promoter. The transgenic DNA fragment was gel-purified and injected into fertilized embryos harvested from C57BL/6J×SJL hybrid mice. Transgenic lines were backcrossed with C57BL/6 J for at least three generations. To generate *Adissp* knockout mice, *Adissp* embryonic stem cell (ESC) clones (in C57BL/6 N background) with conditional potential were obtained from European Mouse Mutant Cell Repository. Germ-line transmissible mice were generated by core facility of UMASS Chan Medical School. These mice were then crossed with *Flp* mice (Stock No. 012930, Jackson Laboratory) to remove the targeting cassette, resulting in exon 3 flanked by loxP sites. The Floxed mice were crossed with *adipnectin*-*cre* mice^33^ to generate adipose-specific *Adissp* knockout (ADKO) mice.

Mice were maintained under a 12 h light/12 h dark cycle at controlled-temperature (23 ± 1 °C) and humidity (50% ± 20%) with free access to food and water. Normal diet containing 4% (w/w) fat and high-fat diet containing 60% (w/w) fat were purchased from Harlan Teklad and Bioserv (#S3282), respectively. Daily food intake of single caged, 2- to 3-month-old male mice was measured for 2 weeks. Fat mass and lean mass were measured by Body Composition Analyzer EchoMRI (Echo Medical Systems). β3-AR agonist CL-316,243 (R&D Systems, #1499) was intraperitoneally injected into mice at 1 mg/kg body weight for 2 days. β-blocker (Sigma, #P0884) was intraperitoneally injected into mice at 25 mg/kg body weight once a day for 7 days. For acute cold exposure, mice were placed at 4 °C with water but without food, and core body temperature was measured with the Microtherma 2 rectal probe (Thermoworks). For studies on thermoneutral condition, mice were maintained under a 12 h light/12 h dark cycle at controlled temperature (29.4 ± 1.6 °C) and humidity (50% ± 20%) with free access to food and water. Whole-body energy expenditure and locomotor activity of adult mice were monitored by a comprehensive lab animal monitoring system (CLAMS) in UMASS Metabolism core. Gender-matched littermate controls were used in all the studies. All the experimental mice were monitored every day or every week, and the body weight was weighed during the experiment. At the end of experiment, the mice were euthanized with CO_2_ inhalation. All animal studies were performed according to procedures approved by the UMASS Medical School’s Institutional Animal Care and Use Committee (IACUC).

### Adenovirus production, purification, and injection

Full-length mouse *Adissp* and human *ADISSP* cDNA were generated by PCR. Adenoviral *Adissp* overexpression plasmids were constructed using AdEasy-1 system^43^. Adenoviruses were purified with cesium chloride ultracentrifugation. All viral titers were predetermined, and same number of viral Plaque Formation Unit (PFU) was used for experimental and control samples.

For tail-vein injections, *GFP*, mouse *Adissp* or human *ADISSP* adenoviruses (1–2 × 10^10^ PFU/mice) were injected in a total volume of 150 μL. To overexpress *Adissp* in BAT, *Adissp* or *GFP* adenoviruses (1 × 10^10^ PFU at the volume of 20 μL per site) were injected into six different sites of bilateral BAT of 3-month-old C57BL/6 J male mice to cover the whole fat pad. To delete *Adissp* in BAT, *Cre* or *GFP* adenoviruses (1 × 10^10^ PFU at the volume of 20 μL per site) were injected into six different sites of bilateral BAT of 6-month-old *Adissp* floxed female mice to cover the whole fat pad. To overexpress *Adissp* in iWAT, *Adissp* and *GFP* adenoviruses (1 × 10^10^ PFU in 30 μL per site) were injected into the left and right iWAT pad of the same C57BL/6J male mice, respectively. Five different sites were injected to cover the whole fat pad. One week after the injection, the mice were dissected and tissues were harvested.

### Gene expression analysis

Total RNA was extracted from cells or tissues using TRIzol reagent (Invitrogen). One microgram total RNA was converted into first-strand complementary DNA (cDNA) with random primers using an IScript cDNA synthesis kit (Invitrogen). qPCR was performed with SYBR green fluorescent Dye (Bio-Rad, #1725272) using an ABI7300 PCR machine, and *36B4* was used as an internal control. Relative mRNA expression was determined by the ΔΔ-Ct method. The sequences of qPCR primers used in this study are listed in Supplementary Table 2.

### Protein extraction and western blotting

Total protein was extracted from cells or adipose tissues by using lysis buffer (100 mM NaCl, 50 mM Tris (pH 7.5), 0.5% Triton X-100, 5% (w/v) glycerol) supplemented with complete protease inhibitor cocktail (Roche, #11836170001), phosphatase inhibitor cocktail (Sigma, #P0044) and phenylmethylsulfonyl fluoride (PMSF) (Thermo Fisher Scientific, #36978). For secretion assay of Adissp*, Adissp* adenovirus-transduced brown adipocytes were cultured in serum-free medium with 1× protein transport inhibitor cocktail (10.6 μM Brefeldin A and 2 μM Monensin) (Fisher scientific, #50-930-9), 10 μM oligomycin (Sigma, #O4876), or 10 μM FCCP (Carbonyl cyanide-4 (trifluoromethoxy) phenylhydrazone) (Sigma, #C2920) for indicated times, and conditioned medium was collected. For secretion assay of Adissp with CL-316,243, *Adissp* adenovirus-transduced brown adipocytes were cultured in serum-free medium with 10 μM CL-316,243. The medium was harvested at indicated time intervals and fresh serum-free medium with 10 μM CL-316,243 was supplied. The medium was concentrated with a 3-kDa cut-off centrifugal filter (Millipore). Samples were subjected to SDS-PAGE under reducing conditions, transferred, and immunoblotted with antibodies against Flag (Sigma, #F7425; 1:2000 dilution), Adissp (MyBioSourse, #MBS1493234; 1:200 dilution), Ucp1 (Sigma, #U6382; 1:1000 dilution), Phospho-PKA Substrate (Cell signaling, #9624; 1:1000 dilution), HSL (Cell Signaling, #4107; 1:1000 dilution), pHSL^S660^ (Cell Signaling, #45804; 1:1000 dilution), Tubulin (DSHB, #E7; 1:5000 dilution), Adiponectin (Cell Signaling, #2789; 1:500 dilution), or Actin (Santa Cruz, #sc-47778; 1:2000 dilution).

For serum samples, Albumin/IgG was removed from 60 μL serum according to the manufacturer’s instructions (Millipore, #122642). The albumin/IgG-depleted serum was concentrated to 60 μL with 10-kDa cut-off centrifugal filter. Thirty microliters serum was subjected to SDS-PAGE under reducing conditions, transferred, and immunoblotted with antibodies against Adissp (MyBioSourse, #MBS1493234, 1:200 dilution).

### Glucose and insulin tolerance tests

For glucose tolerance test, mice were fasted overnight or otherwise indicated. Glucose (2 g/kg body weight) was administered intraperitoneally, and blood glucose was measured at 0, 30, 60, 90, and 120 min. For insulin tolerance test, mice were fasted 5 h. Insulin (0.75 U/kg body weight) was administered intraperitoneally, and blood glucose was measured at 0, 30, 60, 90, and 120 min.

### Oxygen consumption assay

Subcutaneous inguinal WAT depots were weighed and chopped into small pieces. Portions with equal amount (50 mg) were suspended in 5 mL Dulbecco’s phosphate-buffered saline supplemented with 25 mM glucose, 1 mM pyruvate and 2% BSA, and respiration was measured with a biological oxygen monitor (YSI model 5300 A).

### In vivo glucose uptake

In vivo glucose uptake was measured as previously described^44^. Briefly, wild-type mice were tail-vein infused with *GFP* or *Adissp* adenoviruses, and at day 7, mice were fasted for 5 h. *Adissp* floxed and ADKO mice were cold challenged for 3 h without food. ^3^H-2-deoxyglucose (100 μCi/kg body weight) (PerkinElmer) was then intravenous injected. One hour post injection, mice were sacrificed and tissues were collected, weighed and snap frozen in liquid nitrogen. Tissues were digested by incubating in 500 μL of 1 M NaOH at 60 °C for 60 min and neutralized with 500 μL of 1 M HCl. Two hundred microliters of this neutralized solution were added to 1 mL of 6% perchloric acid, vortexed and centrifuged at 13,000 × *g* for 5 min. Eight hundred microliters supernatant was mixed with 5 mL scintillation cocktail and total radioactivity was quantified in c.p.m. by liquid scintillation counting.

### Protein purification and injection

To increase the yield and facilitate the purification of secreted Adissp, we added a signal peptide at the N-terminus of Adissp and a 6×His tag at its C-terminus using pHL-sec vector (Addgene, #99845)^45^. The resulting DNA fragment was cloned into pENTR1A vector (Addgene, #17398). Lentiviral construct was generated via recombination of pENTR1A-*Adissp* into pLenti CMV/TO Puro DEST (Addgene, #17293), and lentiviruses were produced by co-transfection along with plasmids pLP1, pLP2, and pVSVG into HEK293 cells. HEK293 cells were infected with lentiviruses and Adissp expressing stable cells were selected with puromycin. Serum-free medium from stable cells was collected, and incubated with Ni-NTA Agarose (QIAGEN, #30210) at 4 °C for 2 h. The samples were loaded into Poly-Prep chromatography columns (Bio-Rad, # 7311550). The columns were washed with washing buffer (PBS containing 5 mM imidazole (Sigma, #I5513). Adissp protein was eluted with elution buffer (PBS containing 250 mM imidazole), concentrated and then buffered in PBS. Endotoxin level (0.08 EU/mL) was measured using a commercial kit (Thermo Scientific, #88282). Purified Adissp protein was used to treat adipocytes, or intravenously inject into 3-month-old C57BL/6J male mice (1 mg/kg body weight) in a total volume of 150 μL. PBS was used as vehicle control. Adissp protein was used within 1 week after purification.

### Competitive ELISA assay

The competitive ELISA assay was modified from previously described^46^. Wells of microplate (R&D, #DY990) were filled with 50 μL of recombinant Adissp protein (250 ng/mL) in coating buffer (PBS, pH 7.4), with the exception of 2 blank controls filled with coating buffer, and were incubated overnight at 4 °C. They were then washed twice with washing buffer (PBS with 0.05% Tween-20), and filled with 200 μL blocking buffer (PBS with 3% BSA) and incubated for 2 h at room temperature. One hundred and thirty microliters of purified mouse Adissp protein with various concentrations or samples were pre-incubated with Adissp antibody (250 ng/mL) for l h at room temperature. Wells were washed twice with washing buffer and filled with 50 μL per well of pre-incubated standards or samples in duplicate and incubated for 3 h at room temperature. After washed three times with washing buffer, 50 μL of the HRP-conjugated mouse-anti-rabbit antibody (1:5000) was added to all the wells and incubated for 1 h at room temperature. Then they were washed three times with washing buffer, filled with 100 μL TMB substrate solution (Thermo Fisher Scientific, #34028) and incubated for 30 min in the dark at room temperature. The reaction was terminated by the addition of 100 μL stop solution (2 M sulfuric acid) per well. Optical densities at 450 nm were measured on an ELISA plate reader within 10 min. All dilutions were done in the blocking buffer.

### cAMP assay

cAMP assay was done according to the manufacturer’s instructions (R&D Systems, #KGE012). In brief, inguinal WAT depot was weighed, and equivalent amount (~50 mg) was minced with scissors and then homogenized in 500 μL 0.1 N HCl. Lysates were collected and centrifuged at 10,000 g for 10 min. Then 200 μL supernatant was transferred to new tubes and mixed with 28 μL 1 N NaOH and diluted with calibrator diluent RD5-55. Samples were centrifuged again at 10,000 × *g* for 10 min, and 100 μL supernatant was used for assay. To measure cAMP in cell culture, cells were resuspended in cold PBS buffer containing 0.1 N HCl after washed with cold PBS, incubated at room temperature for 10 min and neutralized with 1 N NaOH (1:10). Cell lysates were centrifuged at 600 × *g* for 10 min, diluted twofold with calibrator diluent RD5-55 and 100 μL supernatant was used for assay.

### Adissp binding assay

Recombinant Adissp was purified from conditioned medium of HEK293 cells, and recombinant GFP protein was purified from *E. coli* BL21. Recombinant Adissp and GFP protein was labeled with membrane non-permeable biotin according to the manufacturer’s instructions (Thermo Fisher Scientific, #A39256).

Three-month-old wild-type male mice were challenged with or without cold (4 °C) for 8 h, and then the mice were sacrificed. The inguinal WAT were fixed with 10% formalin overnight and paraffin-embedded. The formalin-fixed and paraffin-embedded tissue sections were deparaffinized and rehydrated by graded concentrations of ethanol solutions. The sections were incubated with biotin-GFP or biotin-Adissp protein (500 ng/ml) at room temperature for 1 h and gently washed by PBS. Next, the tissue sections were incubated with Alexa Fluor 488-conjugated streptavidin (Invitrogen, #S32354, 1:500 dilution) and DAPI (Sigma, #D9542) at room temperature for 2 h and gently washed by PBS. The images were acquired with an inverted Nikon Eclipse Ti2 confocal microscope (Nikon Instruments/Nikon Corp).

In vitro differentiated brown adipocytes were treated with biotin-GFP or biotin-Adissp protein (500 ng/mL) at 37 °C for 1 h. The cells were gently washed by PBS and fixed with 3.7% formalin at 37 °C for 15 min without permeabilization. Subsequently, the cells were incubated with Alexa Fluor 488-conjugated streptavidin (Invitrogen, #S32354, 1:500 dilution) at room temperature for 2 h. Then, the cells were stained with DAPI (Sigma, #D9542) at room temperature for 20 min and gently washed by PBS. The images were acquired with an inverted Nikon Eclipse Ti2 confocal microscope (Nikon Instruments/Nikon Corp).

Adissp tissue binding assay through detecting SEAP activity was performed as previously described^34,47^. Briefly, HEK293 cells were transfected with plasmids expressing SEAP (Addgene, #24595) or SEAP-Adissp. Serum-free medium was collected and concentrated using 10-kDa centrifugal filter. Frozen tissue slices were incubated with SEAP or SEAP-Adissp conditioned medium for 90 min at room temperature. They were fixed in a solution containing 20 mM HEPES (pH 7.4), 60% acetone and 3% formaldehyde after washed four times with PBS containing 0.1% Tween-20. After inactivating endogenous alkaline phosphatase at 65 °C for 30 min, the enzymatic activity derived from the fusion protein was detected using NBT/BCIP substrate (Sigma, #B1911). For competition binding, frozen tissue slices were pre-incubated for 60 min with fivefold concentrated conditioned medium from HEK293 cells expressing Adissp.

### Immunofluorescence staining and histology

Differentiated brown adipocytes were gently washed with DMEM and treated with PBS or CL-316, 243 (10 μM) for 45 min. Cells were then fixed with 3.7% formalin at 37 °C for 15 min. The cells were permeabilized with ice-cold 100% methanol at −20 °C for 10 min and washed twice with PBS. Next, the cells were incubated with blocking buffer (PBS containing 5% normal goat serum and 0.3% Triton X-100) at room temperature for 60 min and incubated with Adissp antibody (1:200 dilution) and β-actin antibody (1:200 dilution) in blocking buffer at 4 °C overnight. After incubation, the cells were gently washed twice with PBS and incubated with Alexa Fluor 488-conjugated (Thermo Fisher, #A-11034, 1:1000) and 594-conjugated secondary antibody (Thermo Fisher, #A-11032, 1:1000) at room temperature for 2 h. Finally, the cells were stained with DAPI (Sigma, #D9542) at room temperature for 20 min, and images were acquired with an inverted Nikon Eclipse Ti2 confocal microscope (Nikon Instruments/Nikon Corp).

Tissues fixed with 10% formalin were paraffin-embedded. H&E staining was performed according to standard procedures. Immunofluorescence staining was done as described previously^48^. Paraffin-embedded tissue sections were deparaffinized and rehydrated through graded ethanol solutions. Slides were incubated with Ucp1 antibody (Sigma, #U6382, 1:500) in dilution buffer (PBS containing 1% BSA and 0.3% Triton X-100) at 4 °C overnight after pre-incubated with blocking buffer (PBS containing 5% normal goat serum and 0.3% Triton X-100) for 1 h. Next, the slides were washed 3 times with PBS and incubated with Alexa Flour 488-conjugated goat anti-rabbit secondary antibody (Thermo Fisher Scientific, #A-11034, 1:1000) for 2 h at room temperature. The slides were then washed 3 times with PBS and stained with 10 μg/mL DAPI (Sigma) for 1 h at room temperature. Images were acquired and processed with the same settings.

### Statistical analysis

Data are presented as mean ± standard error of mean (s.e.m.). For comparisons between two groups of normally distributed data, two-tailed Student’s *t* test were used and performed with Microsoft Excel 2013. For comparisons between multiple groups (for example, GTT and ITT data), two-way repeated measures ANOVA with post hoc test by Fisher’s LSD test were used and performed with Prism 8. To analyze energy expenditure of the mice, analysis of covariance (ANCOVA) was performed with R script and the body weight of each mouse was used as the covariate. *p* < 0.05 was considered statistically significant in all the experiments. The statistical parameters and the number of mice used per experiment are stated in the figure legends.

### Reporting summary

Further information on research design is available in the Nature Portfolio Reporting Summary linked to this article.

## Supplementary information

Supplementary Information

Reporting Summary

## Data Availability

The data supporting the findings of this study are available within the Supplementary Information file and Source Data file. All the RNA-seq and Microarray data used in this study were published. RNA-Seq data used in Fig. 1a was published^13^ and is accessible in Gene Expression Omnibus (GEO) (accession number GSE56367); RNA-Seq data shown in Fig. 1e were obtained from supplementary data of published work [https://bmcbiol.biomedcentral.com/articles/10.1186/s12915-019-0693-x#availability-of-data-and-materials]^18^; RNA-Seq data shown in Fig. 1g were published^19^ and the primary data are available through the Array Express Archive with accession number E-MTAB-1733; microarray data shown in Fig. 1h was published^20^ and is accessible in GEO (GSE2508); microarray data used in Fig. 1i was published^21^ and is accessible in GEO (GSE70353). Proteomics data of proteins secreted from brown adipocytes are available via ProteomeXchange with identifier PXD038041. Source data are provided with this paper.

## Source data

Source Data
