# Supplementary material for: A brown fat-enriched adipokine Adissp controls adipose thermogenesis and glucose homeostasis
Source: Nat Commun. 2022 Dec 10;13:7633. doi: 10.1038/s41467-022-35335-w (PMC9741603; doi:10.1038/s41467-022-35335-w)

Supplementary Table 1

BAT-enriched proteins that are also present in conditioned medium of brown adipocyte culture.

Gene	Description
Aldoa	aldolase A, fructose-bisphosphate
Prkar2b	protein kinase, cAMP dependent regulatory, type II beta
Apoc1	apolipoprotein C-I
Tkt	transketolase
Agpat2	1-acylglycerol-3-phosphate O-acyltransferase 2 (lysophosphatidic acid acyltransferase, beta)
Thrsp	thyroid hormone responsive
Acly	ATP citrate lyase
Mecr	mitochondrial trans-2-enoyl-CoA reductase
Taldo1	transaldolase 1
Acadl	acyl-Coenzyme A dehydrogenase, long-chain
Hadhb	hydroxyacyl-Coenzyme A dehydrogenase/3-ketoacyl-Coenzyme A thiolase/enoyl-Coenzyme A hydratase (trifunctional protein), beta subunit
Echs1	enoyl Coenzyme A hydratase, short chain, 1, mitochondrial
Hadh	hydroxyacyl-Coenzyme A dehydrogenase
Hibch	3-hydroxyisobutyryl-Coenzyme A hydrolase
Decr1	2,4-dienoyl CoA reductase 1, mitochondrial
Acsl1	acyl-CoA synthetase long-chain family member 1
Etfa	electron transferring flavoprotein, alpha polypeptide
Slc16a1	solute carrier family 16 (monocarboxylic acid transporters), member 1
Acadm	acyl-Coenzyme A dehydrogenase, medium chain
Dhrs4	dehydrogenase/reductase (SDR family) member 4
Nudt7	nudix (nucleoside diphosphate linked moiety X)-type motif 7

(continued)

Gene	Description
Ddt	D-dopachrome tautomerase
Atpaf2	ATP synthase mitochondrial F1 complex assembly factor 2
Eci1	enoyl-Coenzyme A delta isomerase 1
Gpd1	glycerol-3-phosphate dehydrogenase 1 (soluble)
Bckdha	branched chain ketoacid dehydrogenase E1, alpha polypeptide
Aifm1	apoptosis-inducing factor, mitochondrion-associated 1
Zadh2	zinc binding alcohol dehydrogenase, domain containing 2
Acads	acyl-Coenzyme A dehydrogenase, short chain
Dlst	dihydrolipoamide S-succinyltransferase (E2 component of 2-oxo-glutarate complex)
Acaca	acetyl-Coenzyme A carboxylase alpha
**1700037H04Rik**	**RIKEN cDNA 1700037H04 gene**
Pfkl	phosphofructokinase, liver, B-type
Acat2	acetyl-Coenzyme A acetyltransferase 2
Uck1	uridine-cytidine kinase 1
Fam82a2	Rmdn3
Eno1	enolase 1, alpha non-neuron
Ahcyl1	S-adenosylhomocysteine hydrolase-like 1
Coasy	Coenzyme A synthase
Gngt2	guanine nucleotide binding protein (G protein), gamma transducing activity polypeptide 2
Acaa2	acetyl-Coenzyme A acyltransferase 2 (mitochondrial 3-oxoacyl-Coenzyme A thiolase)
4931406C07Rik	RIKEN cDNA 4931406C07 gene

Supplementary Table 2

Primer sequences used for quantitative PCR in this study.

Gene	Forward	Reverse
36B4	GCGACCTGGAAGTCCAACTAC	ACGTTGTCTGCTCCCACAAT
Ucp1	GGATTGGCCTCTACGACTCA	TGCCACACCTCCAGTCATTA
Cidea	CGGGTAGTAAGTATGTCCCA	CAGCATAGGACATAAACCTCA
Cox7a1	CAGCGTCATGGTCAGTCTGT	AGAAAACCGTGTGGCAGAGA
Cox8b	GAACCATGAAGCCAACGACT	GCGAAGTTCACAGTGGTTCC
CytC	GCAAGCATAAGACTGGACCAAA	TTGTTGGCATCTGTGTAAGAGAATC
mCpt1	GGGCACCTCTGGGAGTTTGT	TTGGCTCACCCACACAGTGT
Pgc1α	AGCCGTGACCACTGACAACGAG	GCTGCATGGTTCTGAGTGCTAAG
aP2	GGCGTGACTTCCACAAGAGTTTA	GCCTCTTCCTTTGGCTCATG
Adissp	TGACTCGGTGGTCATGGTTA	ACGGATGTCCTTGCTCAGTC
Gpi1	AAGGAGGTGATGCAGATGCT	GCCCGATTCTCGGTGTAGT
Hk2	TGATCGCCTGCTTATTCACGG	AACCGCCTAGAAATCTCCAGA
Pfkp	CGCCTATCCGAAGTACCTGGA	CCCCGTGTAGATTCCCATGC
Pkm2	GCCGCCTGGACATTGACTC	CCATGAGAGAAATTCAGCCGAG
Fasn	TGATGATTCAGGGAGTGGATATTG	CCGAGCCAGGGACTTCTTAGT
Cpt1a	TCATCCATGCATACCAAAGTG	ACGCCACTCACGATGTTCTT
Pparα	ACAAGGCCTCAGGGTACCA	GCCGAAAGAAGCCCTTACAG
Lcad	CCCTCCGCCCGATGTT	AAGGAGTTTCTAGACGCGCTTCT
Vlcad	GCCAGGGCAGAATCGAAGT	TGGTAAGCTGGCCTTTGAACAT
ADISSP	TACTCGGCGCACAAAGAGG	CCACACACTTGACACCATCCA

Supplementary Figure 1

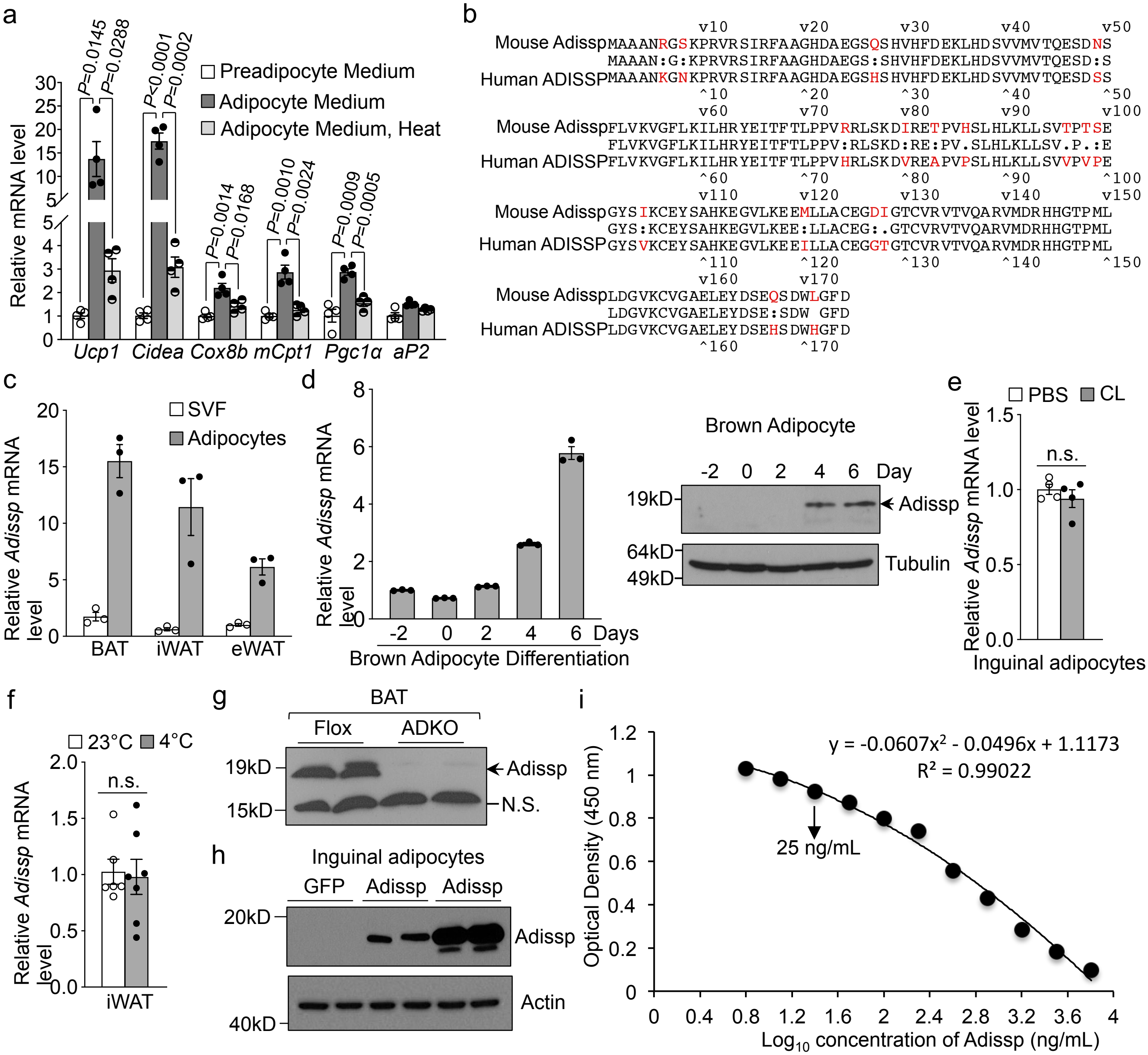

Supplementary Figure 1 | Identification of Adissp (Adipose-secreted signaling protein). a, Gene expression in inguinal adipocytes treated with indicated medium for 6 days (n=4). **b**, Alignment of mouse Adissp and human ADISSP protein sequences. **c**, *Adissp* mRNA expression in stromal vascular fraction (SVF) and mature adipocyte fraction (n=3 mice). **d**, *Adissp* mRNA (n=3) and protein levels during brown adipogenesis. **e**, *Adissp* expression in primary inguinal adipocytes treated with 10 μM CL-316,243 for 3 hours (n=4). **f**, *Adissp* expression in iWAT from WT male mice housed at 23°C (n=6 mice) or 4°C (n=7 mice) for 6 hours. **g**, Western blot of Adissp in BAT from *Adissp* adipose tissue knockout mice (ADKO) and Flox controls. N.S., non-specific band. Two times experiments were repeated independently with similar results. **h**, Western blot of Adissp in inguinal adipocytes transduced with *GFP*, *Adissp* or *ADISSP* adenoviruses. Two times experiments were repeated independently with similar results. **i**, Standard curve of the competitive ELISA assay for Adissp. Data are mean ± s.e.m. P values were determined by two-tailed Student's t test, n.s. (not significant).

Supplementary Figure 2

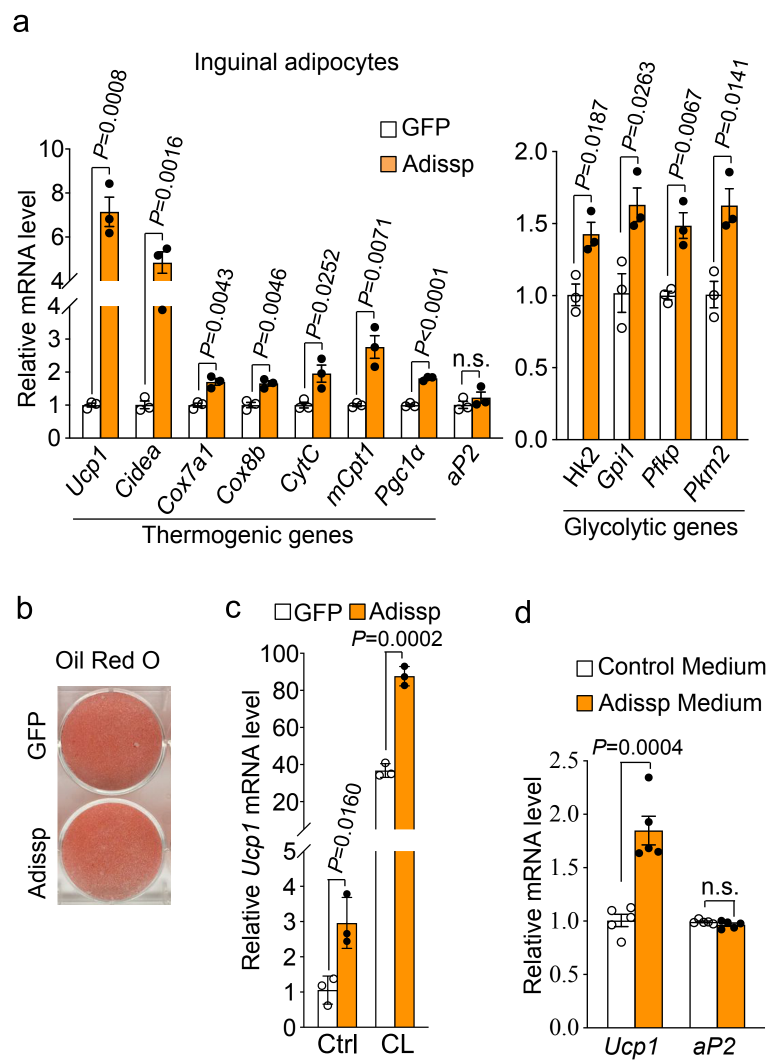

Supplementary Figure 2 | Adissp induces thermogenic gene expression in adipocytes. a, Gene expression analysis in primary inguinal adipocytes transduced with *Adissp* or *GFP* adenoviruses (n=3). **b**, Oil Red O staining of inguinal adipocytes in (**a**). **c**, Gene expression analysis in inguinal adipocytes transduced with *Adissp* or *GFP* adenoviruses (n=3) treated with PBS (Ctrl) or 10 μM CL-316,243 (CL) for 3 hours. **d**, Gene expression analysis in inguinal adipocytes treated with indicated conditioned medium for 6 days (n=5). Data are mean ± s.e.m. P values were determined by two-tailed Student’s t test, n.s. (not significant).

Supplementary Figure 3

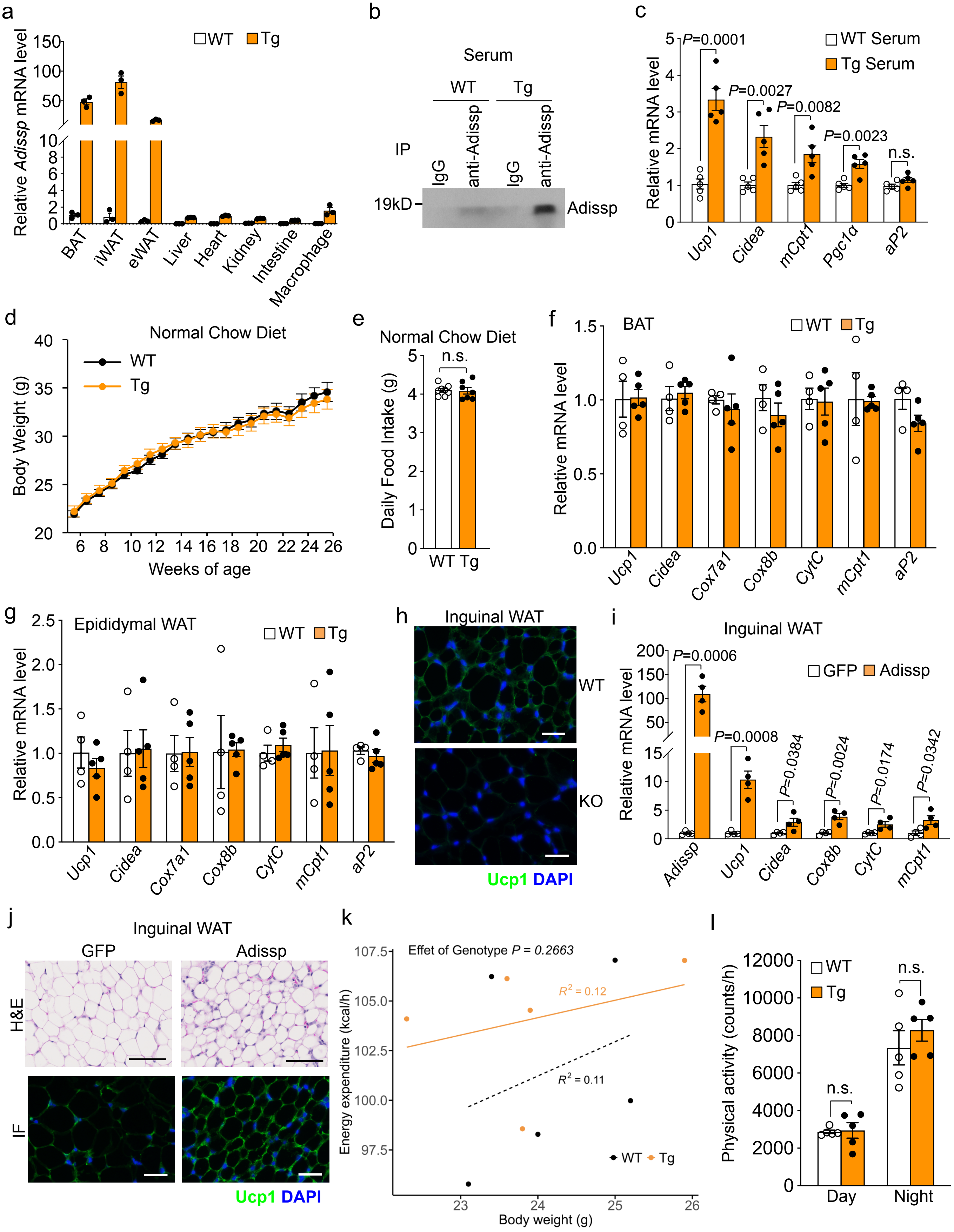

Supplementary Figure 3 | Phenotypes of *Adissp* transgenic mice. a, Relative mRNA levels of *Adissp* in different tissues from *Adissp* transgenic (Tg) mice and wild-type (WT) littermate controls (n=3 mice per group). **b**, Detection of Adissp in WT and *Adissp* Tg mice serum after immunoprecipitation with Adissp antibody. This experiment was done one time. **c**, Gene expression analysis in inguinal adipocytes treated with 10% serum from *Adissp* Tg mice or WT controls (n=5). **d**, Body weight of *Adissp* Tg mice (n=9) and WT controls (n=6) on normal chow diet. **e**, Daily normal chow food intake of 3-month-old male *Adissp* Tg mice (n=7) and WT controls (n=8). **f**, **g**, Gene expression analysis in BAT (**f**) and epididymal WAT (**g**) from 2-month-old male *Adissp* Tg mice (n=5) and littermate controls (n=4). **h**, Ucp1 immunofluorescence staining of inguinal WAT from 3-month-old male WT and *Ucp1* knockout mice (n=3 mice per group). **i**, Gene expression analysis in inguinal WAT injected with *Adissp* adenovirus (n=4 mice per group). **j**, H&E and Ucp1 immunofluorescence staining of inguinal WAT injected with *Adissp* adenovirus (n=3 mice per group). **k**, ANCOVA analysis of energy expenditure of *Adissp* Tg and WT mice fed on 2 weeks of HFD (n=5 mice per group). ANCOVA analysis with energy expenditure as the dependent variable, body weight as a covariate and genotype as a fixed factor. **l**, Physical activity of mice from (**k**).Scale bar, 200 μm. Data are mean ± s.e.m. P values were determined by two-tailed Student's t test (**c**, **e**, **f**, **g**, **i** and **l**) or ANCOVA analysis (**k**). n.s. (not significant).

Supplementary Figure 4

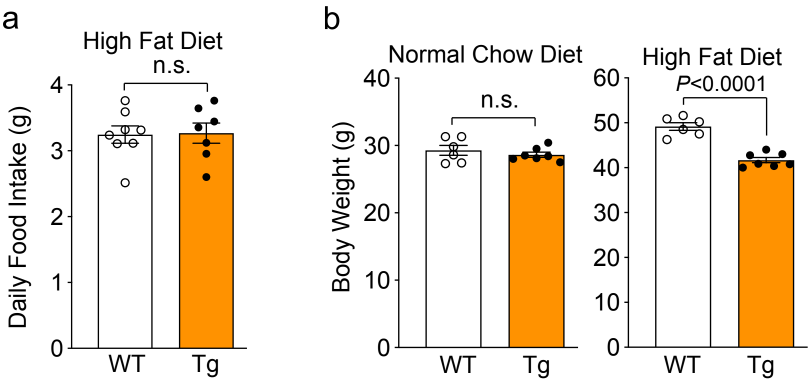

Supplementary Figure 4 | Food intake and body weight of *Adissp* transgenic mice fed on HFD. a, Daily HFD food intake of *Adissp* Tg mice (n=7) and littermate controls (n=8). **b**, Body weight of a second cohort of male *Adissp* Tg mice (n=7) and littermate controls (n=6) before and after fed on 18-week of HFD. Data are mean ± s.e.m. P values were determined by two-tailed Student's t test, n.s. (not significant).

Supplementary Figure 5

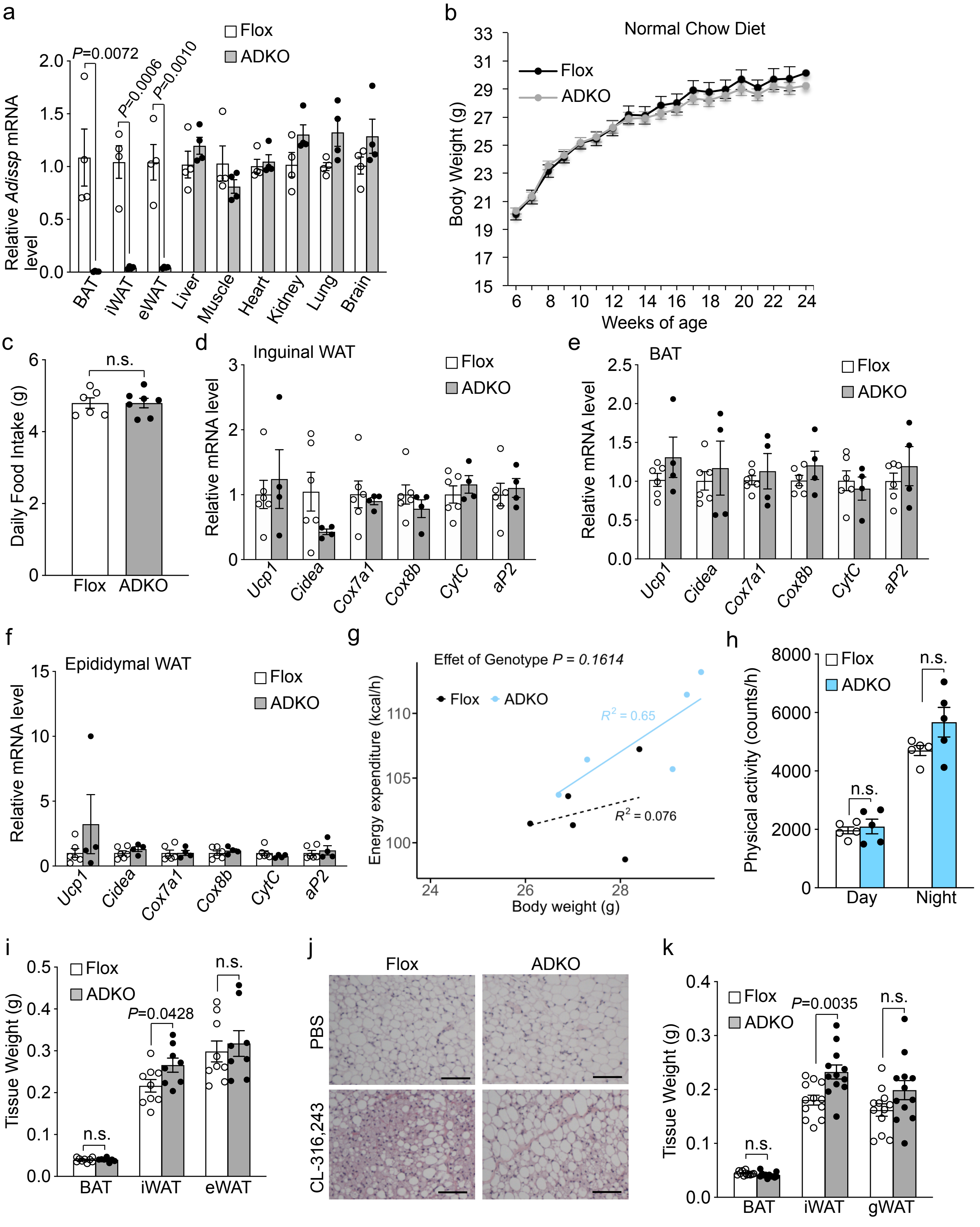

Supplementary Figure 5 | Phenotypes of *Adissp* adipose specific knockout mice. **a**, *Adissp* expression in different tissues of 3-month-old male *Adissp* ADKO mice and Flox controls (n=4). **b**, Body weight of male *Adissp* ADKO mice (n=11) and Flox controls (n=6) on normal chow diet. **c**, Daily normal chow food intake of male *Adissp* ADKO mice (n=7) and Flox controls (n=6). **d-f**, Gene expression analysis of iWAT (**d**), BAT (**e**) and epididymal WAT (**f**) from 3-month-old male *Adissp* ADKO mice (n=4) and Flox littermate controls (n=6) housed at 23 °C. **g**, ANCOVA analysis of energy expenditure of *Adissp* Flox and ADKO mice fed on 3 weeks of HFD (n=5 mice per group). ANCOVA analysis with energy expenditure as the dependent variable, body weight as a covariate and genotype as a fixed factor. **h**, Physical activity of mice from (**g**). **i**, Fat mass of 5-month-old female *Adissp* ADKO mice (n=8) and Flox controls (n=9) after 7 hours cold exposure. **j**, Representative images of H&E staining of iWAT from 3-month-old female *Adissp* ADKO mice and Flox controls after 2 days CL-316,243 administration (n=3 mice per group). **k**, Fat mass of mice from (**j**), (n=12). Scale bar, 200 μm. Data are mean ± s.e.m. P values were determined by two-tailed Student's t test (**a**, **c**, **d-f**, **h**, **i** and **k**) or ANCOVA analysis (**g**), n.s. (not significant).

Supplementary Figure 6

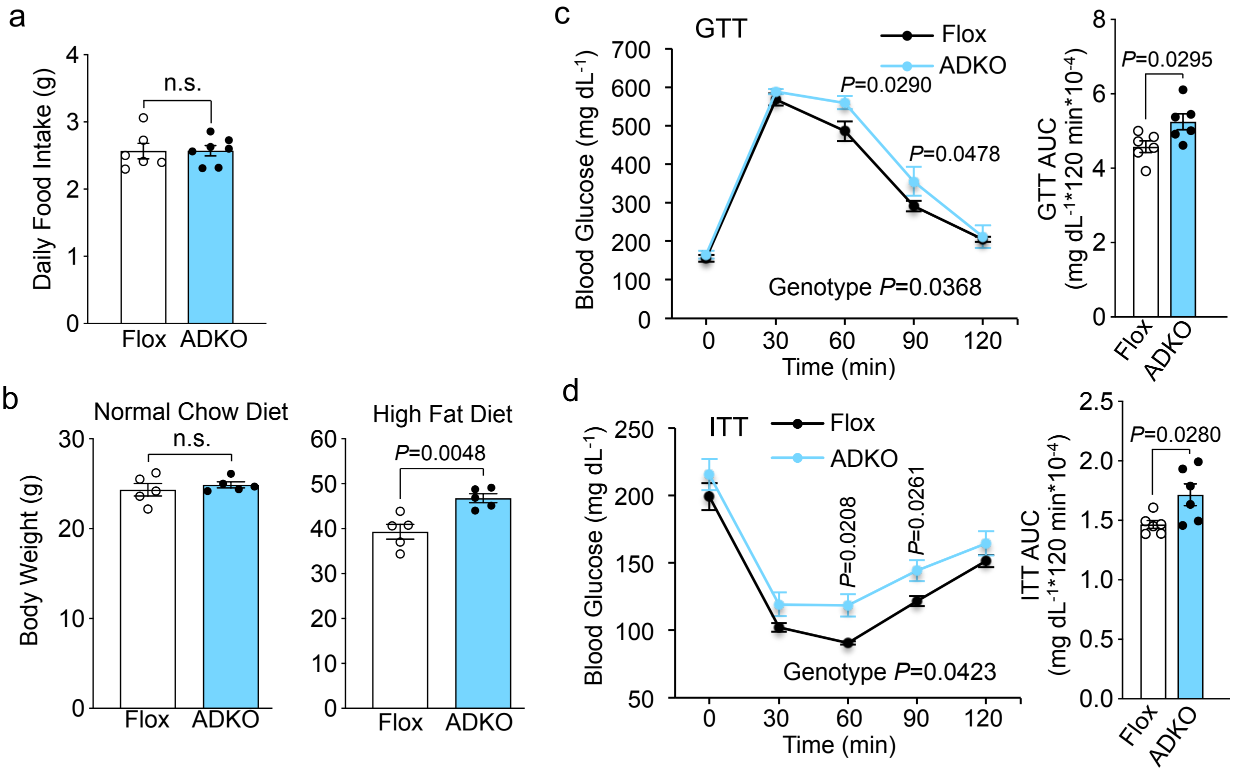

Supplementary Figure 6 | *Adissp* ADKO mice had impaired glucose and insulin tolerance on HFD prior to body weight divergence from that of wild type mice. a, Daily HFD food intake of male *Adissp* ADKO mice (n=7) and Flox controls (n=6). **b**, Body weight of a second cohort of male *Adissp* ADKO mice and Flox controls (n=5 mice per group) before and after fed on 18 weeks of HFD. **c**, GTT of *Adissp* Flox and ADKO mice fed on 5 weeks of HFD (n=6 mice per group). **d**, ITT of *Adissp* Flox and ADKO mice fed on 6 weeks of HFD (n=6 mice per group). Data are mean ± s.e.m. P values were determined by two-tailed Student's t test (**a**, **b** and AUC in **c** and **d**) or two-way repeated measures ANOVA with post hoc test by Fisher's LSD test (**c** and **d**). n.s. (not significant).

Supplementary Figure 7

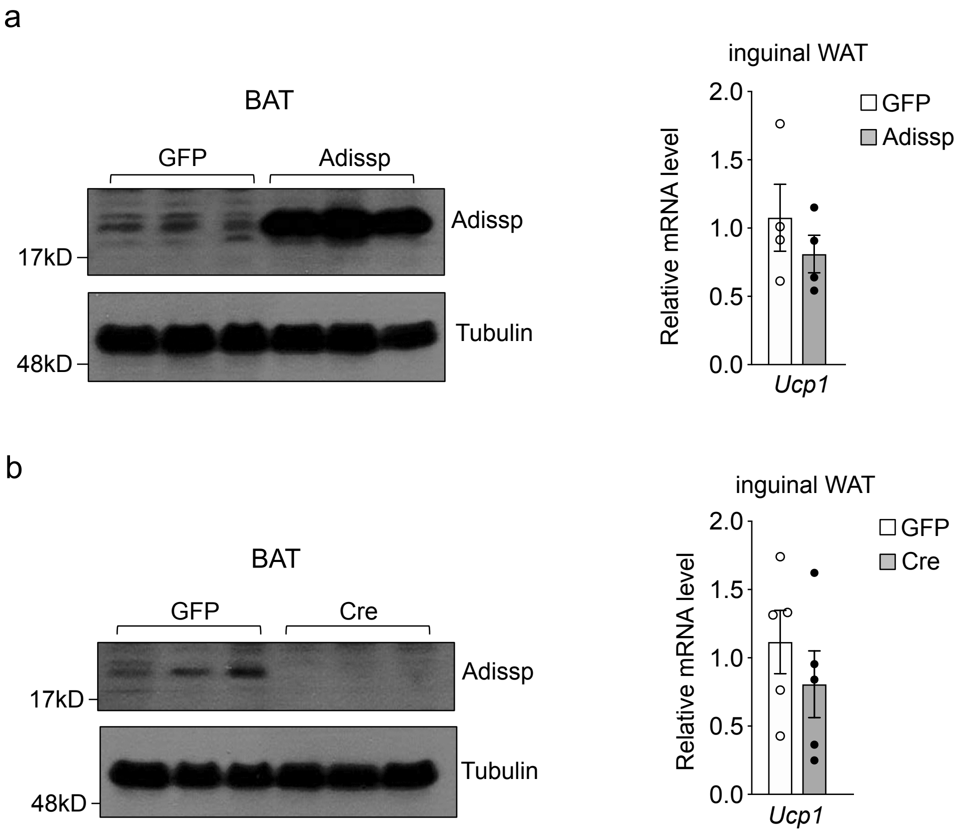

Supplementary Figure 7 | BAT-specific overexpression and deletion of *Adissp* had no effect on *Ucp1* expression in iWAT. a, Western blot of Adissp in BAT and *Ucp1* expression in iWAT (n=4 mice per group) from BAT specific *Adissp* overexpression mice. **b**, Western blot of Adissp in BAT and *Ucp1* expression in iWAT (n=5 mice per group) from BAT specific *Adissp* deletion mice. Data are mean ± s.e.m.

Supplementary Figure 8

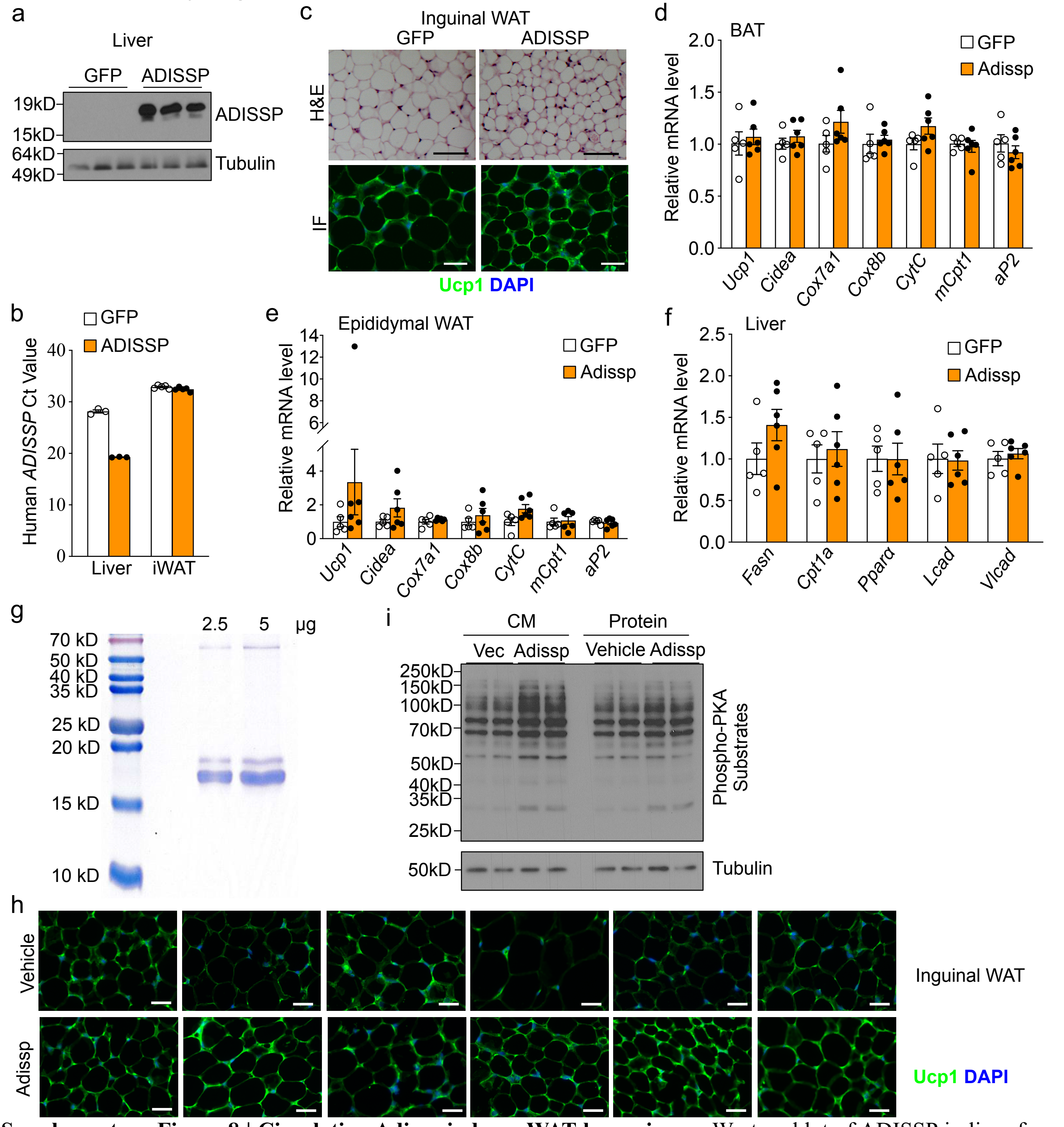

Supplementary Figure 8 | Circulating Adissp induces WAT browning. a, Western blot of ADISSP in liver from mice injected with *GFP* or human *ADISSP* adenoviruses (n=3 mice per group). **b**, *ADISSP* RT-qPCR Ct value in liver (n=3) and inguinal WAT (n=5) from mice injected with *GFP* or *Adissp* adenoviruses. **c**, Representative images of H&E staining and Ucp1 immunofluorescence staining of iWAT from mice injected with *GFP* or *Adissp* adenoviruses (n=3 mice per group). Scale bar, 200 µm. **d-f**, Gene expression analysis in BAT(**d**), eWAT (**e**) and liver (**f**) from mice injected with *GFP* or *Adissp* adenoviruses (GFP, n=5; Adissp, n=6). **g**, SDS-PAGE gel of purified recombinant Adissp protein. Three times experiments were repeated independently with similar results. **h**, Ucp1 immunofluorescence staining of iWAT from mice injected with Adissp protein or vehicle for 9 days (n=7 mice per group). Scale bar, 200 µm. **i**, Western blot analysis of phosphorylated PKA substrates in brown adipocytes treated with conditioned medium (CM) containing 50 nM Adissp protein or 50 nM purified Adissp protein for 45 min. Two times experiments were repeated independently with similar results. Data are mean ± s.e.m.

Supplementary Figure 9

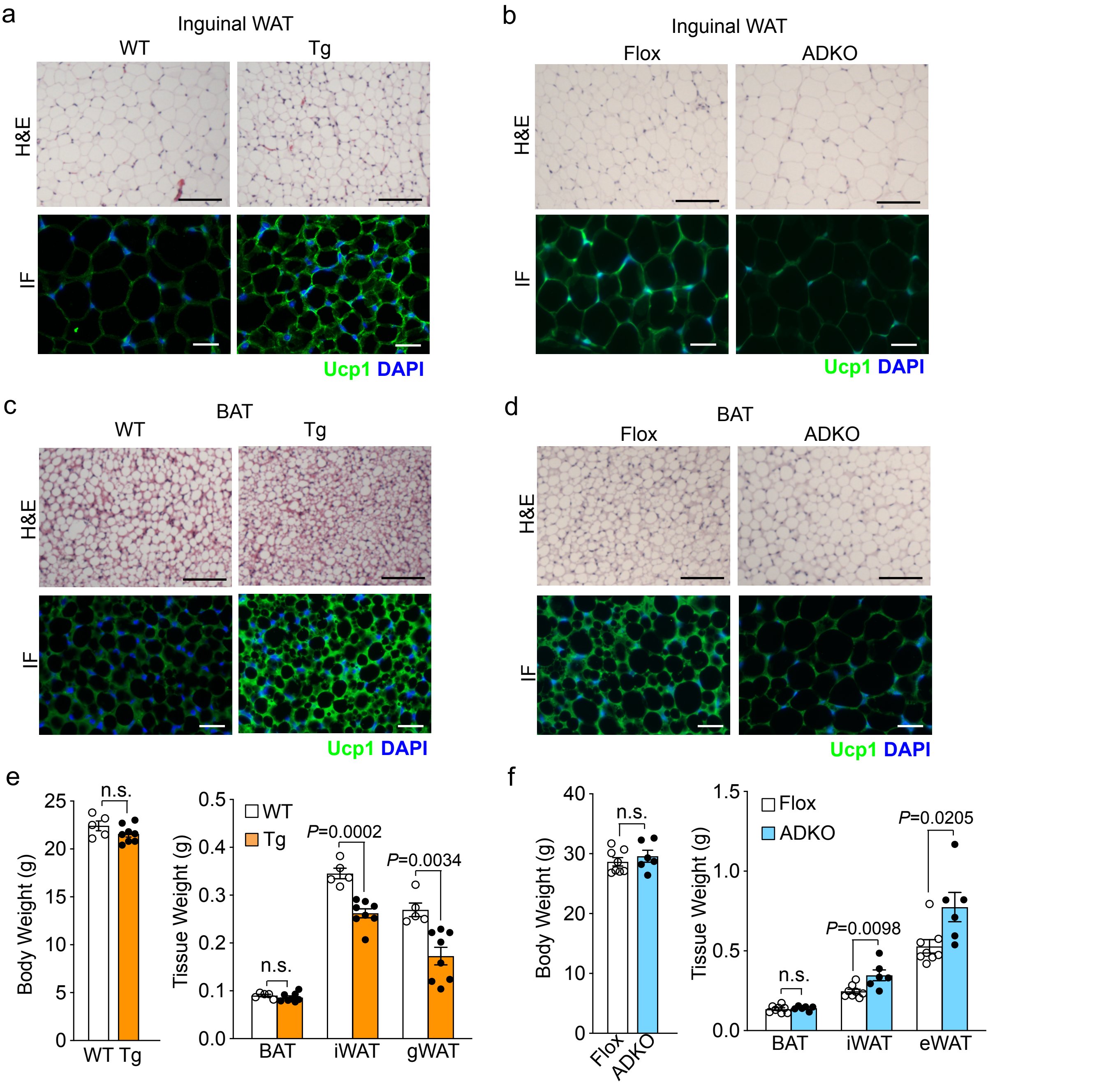

Supplementary Figure 9 | Adissp functions independently of β-AR signaling. a, **b**, H&E staining and Ucp1 immunofluorescence staining of inguinal WAT from *Adissp* Tg female mice and WT controls housed at 30°C for 1 month (**a**) and from *Adissp* ADKO male mice and Flox controls housed at 30°C for 2 months (**b**) (n=3 mice per group). **c**, **d**, H&E staining and Ucp1 immunofluorescence staining of interscapular BAT from *Adissp* Tg female mice and WT controls housed at 30°C for 1 month (**c**) and from *Adissp* ADKO male mice and Flox controls housed at 30°C for 2 months (**d**) (n=3 mice per group). **e**, Body weight and fat mass of *Adissp* Tg female mice (n=8) and WT controls (n=5) housed at 30°C for 1 month. **f**, Body weight and fat mass of *Adissp* ADKO male mice (n=6) and Flox controls (n=8) housed at 30°C for 2 months. Scale bar, 200 µm. Data are mean ± s.e.m. P values were determined by two-tailed Student's t test, n.s. (not significant).

Supplementary Figure 10

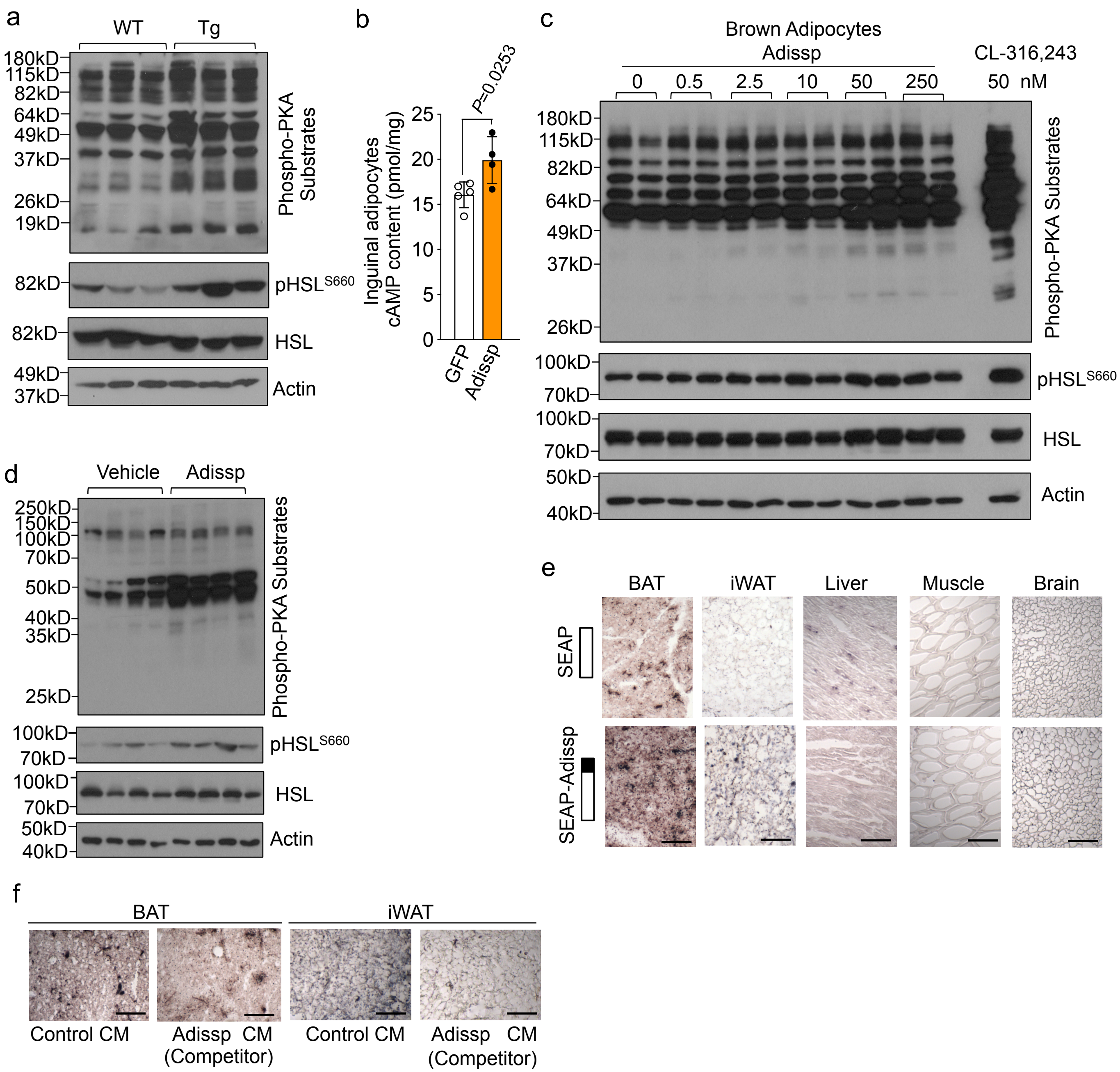

Supplementary Figure 10 | Adissp functions though PKA signaling pathway. a, Western blot analysis of phosphorylated PKA substrates, phospho- and total HSL in iWAT from *Adissp* Tg mice and littermate controls housed at 23°C (n=3 mice per group). **b**, cAMP levels in primary inguinal adipocytes transduced with *Adissp* (n=4) or *GFP* (n=5) adenoviruses. **c,** Western blot analysis of phosphorylated PKA substrates, phospho- and total HSL in brown adipocytes treated with different dose of Adissp protein for 30 min. Two times experiments were repeated independently with similar results. **d**, Western blot analysis of phosphorylated PKA substrates, phospho- and total HSL in iWAT from 3-month-old WT male mice after 9 days of Adissp administration (n=4 mice per group). **e**, SEAP or SEAP-Adissp binding on indicated frozen tissue sections. Scale bar, 200 µm. Two times experiments were repeated independently with similar results. **f**, SEAP-Adissp binding to BAT and iWAT frozen sections in the presence of indicated conditioned medium (CM). Scale bar, 200 µm. Two times experiments were repeated independently with similar results. Data are mean ± s.e.m. P values were determined by two-tailed Student's t test.

Uncropped scans of blots and gels

Supplementary Figure 1d

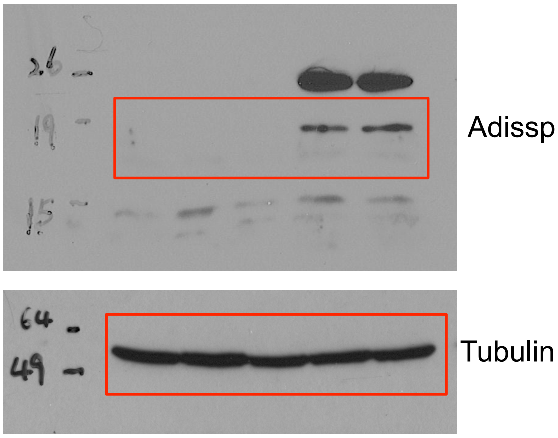

Supplementary Figure 1g

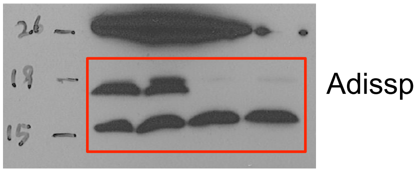

Supplementary Figure 1h

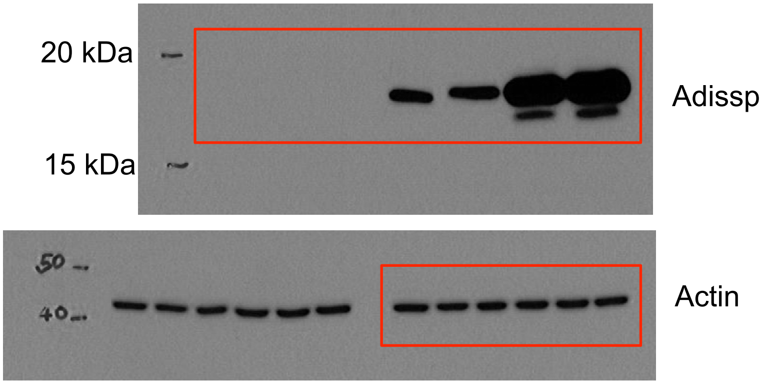

Supplementary Figure 3b

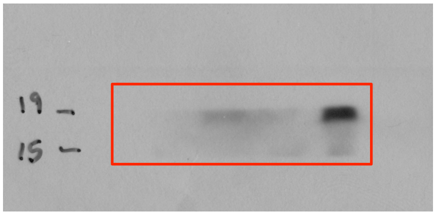

Adissp

Supplementary Figure 7a

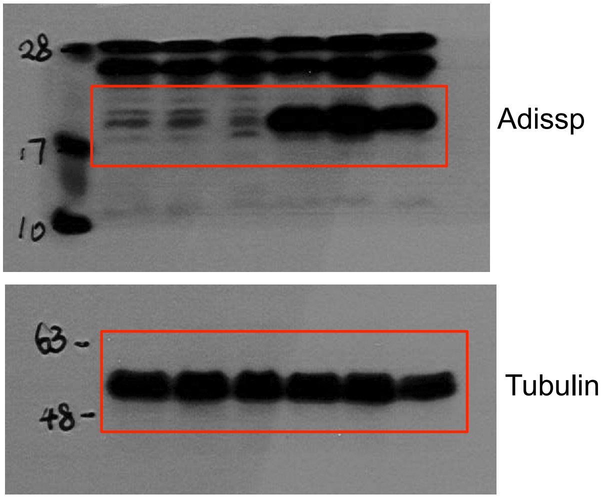

Supplementary Figure 7b

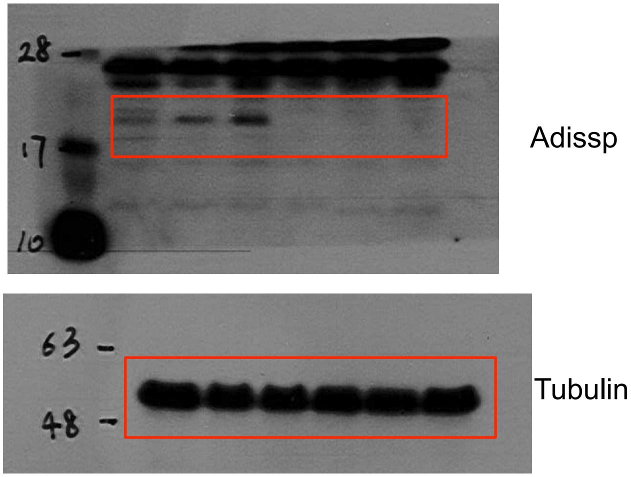

Supplementary Figure 8a

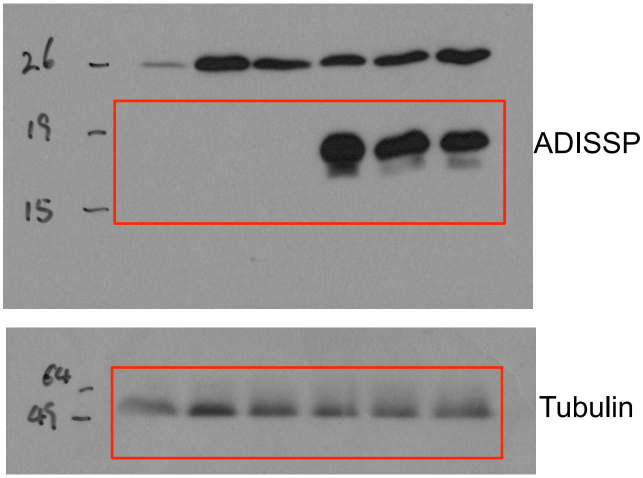

Supplementary Figure 8g

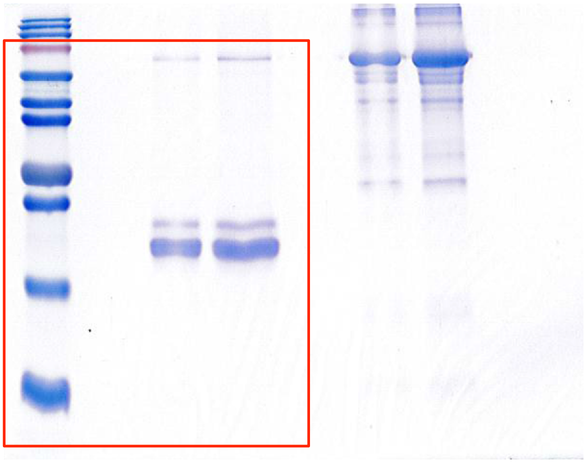

Supplementary Figure 8i

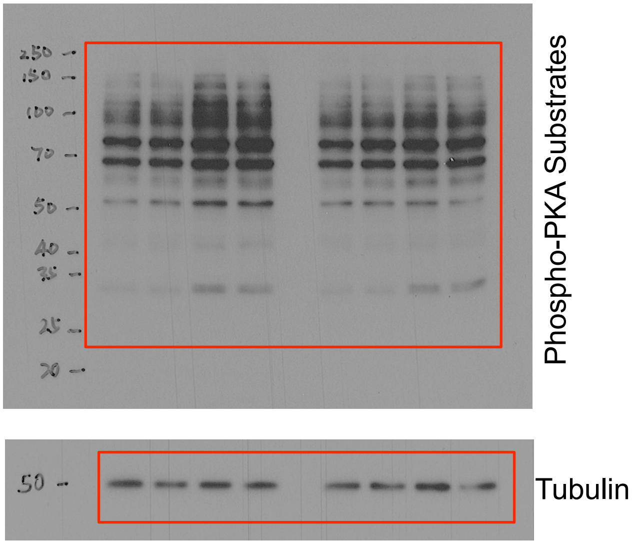

Supplementary Figure 10a

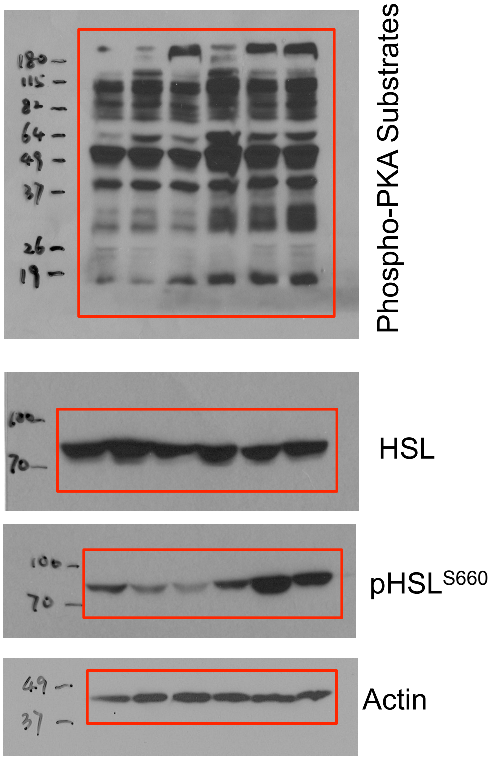

Supplementary Figure 10c

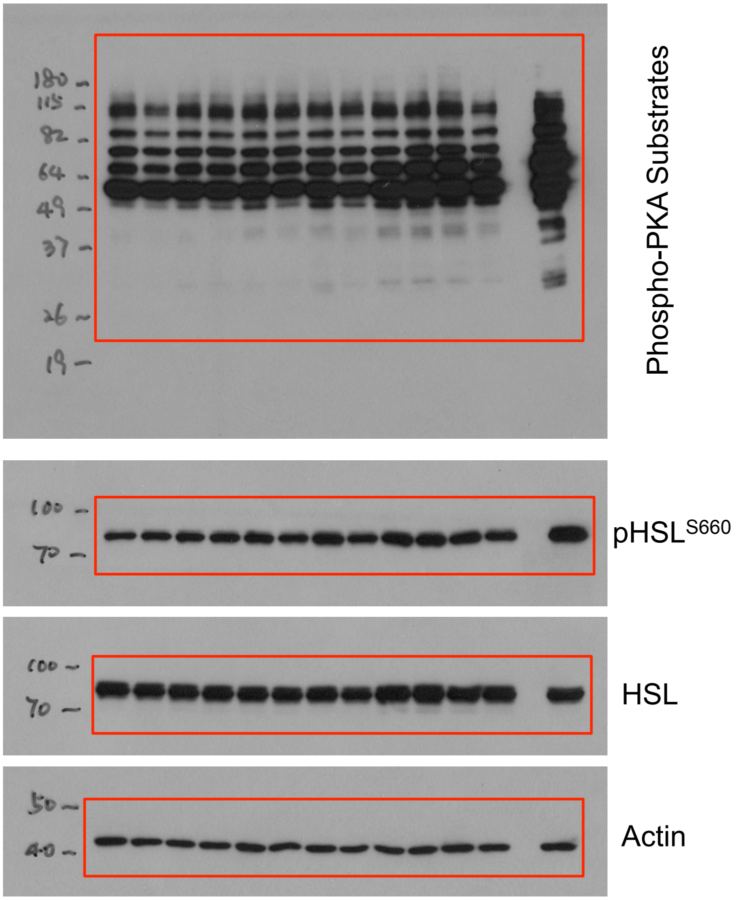

Supplementary Figure 10d

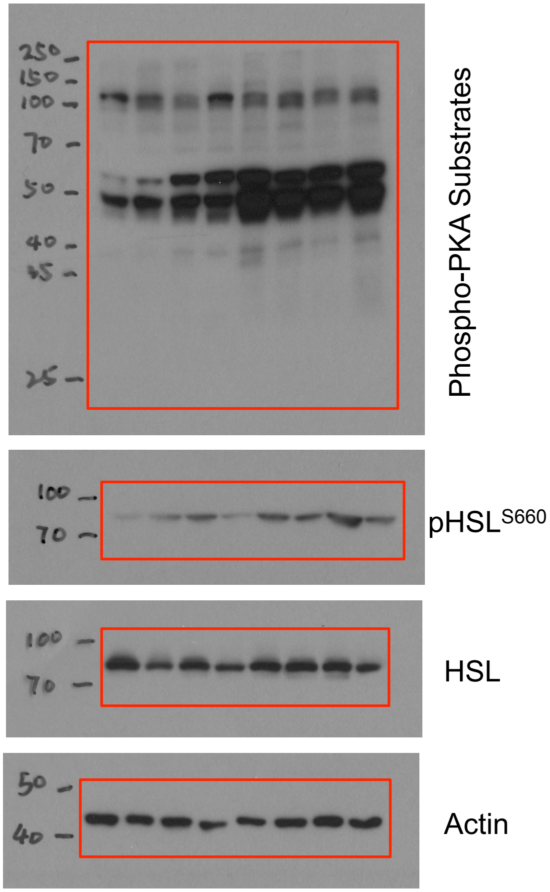

Supplement: Supplementary file 1 — Supplementary Information [file 41467_2022_35335_MOESM1_ESM.pdf]
