# Supplementary material for: A brown fat-enriched adipokine Adissp controls adipose thermogenesis and glucose homeostasis
Source: Nat Commun. 2022 Dec 10;13:7633. doi: 10.1038/s41467-022-35335-w (PMC9741603; doi:10.1038/s41467-022-35335-w)
Supplement: Supplementary file 2 — Reporting Summary [file 41467_2022_35335_MOESM2_ESM.pdf]

nature portfolio

Corresponding author(s): Yong-Xu Wang

Last updated by author(s): Nov 17, 2022

Reporting Summary

Nature Portfolio wishes to improve the reproducibility of the work that we publish. This form provides structure for consistency and transparency in reporting. For further information on Nature Portfolio policies, see our Editorial Policies and the Editorial Policy Checklist.

Statistics

For all statistical analyses, confirm that the following items are present in the figure legend, table legend, main text, or Methods section.

n/a	Confirmed	
☐	☒	The exact sample size (*n*) for each experimental group/condition, given as a discrete number and unit of measurement
☐	☒	A statement on whether measurements were taken from distinct samples or whether the same sample was measured repeatedly
☐	☒	The statistical test(s) used AND whether they are one- or two-sided<br>*Only common tests should be described solely by name; describe more complex techniques in the Methods section.*
☐	☒	A description of all covariates tested
☐	☒	A description of any assumptions or corrections, such as tests of normality and adjustment for multiple comparisons
☐	☒	A full description of the statistical parameters including central tendency (e.g. means) or other basic estimates (e.g. regression coefficient) AND variation (e.g. standard deviation) or associated estimates of uncertainty (e.g. confidence intervals)
☐	☒	For null hypothesis testing, the test statistic (e.g. *F*, *t*, *r*) with confidence intervals, effect sizes, degrees of freedom and *P* value noted<br>*Give P values as exact values whenever suitable.*
☒	☐	For Bayesian analysis, information on the choice of priors and Markov chain Monte Carlo settings
☒	☐	For hierarchical and complex designs, identification of the appropriate level for tests and full reporting of outcomes
☒	☐	Estimates of effect sizes (e.g. Cohen's *d*, Pearson's *r*), indicating how they were calculated

Our web collection on statistics for biologists contains articles on many of the points above.

Software and code

Policy information about availability of computer code

Data collection	QPCR: Applied Biosystems 7300 Real-Time PCR System SDS Version 1.4
Data analysis	SignalP 5.0; SecretomeP-2.0; Microsoft office Excel 2013; GraphPad Prism 8; R script

For manuscripts utilizing custom algorithms or software that are central to the research but not yet described in published literature, software must be made available to editors and reviewers. We strongly encourage code deposition in a community repository (e.g. GitHub). See the Nature Portfolio guidelines for submitting code & software for further information.

Data

Policy information about availability of data

All manuscripts must include a data availability statement. This statement should provide the following information, where applicable:

- Accession codes, unique identifiers, or web links for publicly available datasets
- A description of any restrictions on data availability
- For clinical datasets or third party data, please ensure that the statement adheres to our policy

Proteomics data of proteins secreted from brown adipocytes are available via ProteomeXchange with identifier PXD038041. All the RNA-seq and Microarray data used in this study were published. RNA-Seq data used in Figure 1a was published by Pan et al. [13] and is accessible in Gene Expression Omnibus (GEO) using accession number GSE56367; RNA-Seq data showed in Figure 1e was published and is accessible in the additional file 5 of Xu et al. [18]; RNA-Seq data showed in Figure 1g was published by Fagerberg et al. [19] and the primary data are available through the Array Express Archive (www.ebi.ac.uk/arrayexpress/) under the accession number: E-MTAB-1733; Microarray data showed in Figure 1h was published by Lee et al. [20] and is accessible using accession number GSE2508 and

Microarray data used in Figure 1i was published by Civelek et al. [21] and is accessible using accession number GSE70353.

Human research participants

Policy information about studies involving human research participants and Sex and Gender in Research.

Reporting on sex and gender	N/A
Population characteristics	N/A
Recruitment	N/A
Ethics oversight	N/A

Note that full information on the approval of the study protocol must also be provided in the manuscript.

Field-specific reporting

Please select the one below that is the best fit for your research. If you are not sure, read the appropriate sections before making your selection.

☒ Life sciences ☐ Behavioural & social sciences ☐ Ecological, evolutionary & environmental sciences

For a reference copy of the document with all sections, see nature.com/documents/nr-reporting-summary-flat.pdf

Life sciences study design

All studies must disclose on these points even when the disclosure is negative.

Sample size	For in vivo and in vitro studies, sample sizes were determined based on our previous experiments and experience, and common practices in the research fields (e.g. PMID: 25145289; PMID: 24995979; PMID: 32615086; PMID: 24439384 and PMID: 30122557 etc) using similar methodologies and are sufficient to account for any biological/technical variability. Sample numbers were described in the figures legends.
Data exclusions	No data were excluded.
Replication	Except supplementary Figure 3b, all the biological experiments were repeated, at least, twice with similar results. Liquid Chromatography with tandem mass spectrometry (LC-MS/MS) was performed once but was further validated by western blotting. Western blotting data were confirmed by two or three independent samples.
Randomization	For all the animal experiments, mice were randomly assigned at the time of weaning to minimize any potential bias. For all the cell experiments, equal number of cells were used and all cells in each experiment were from the same pool of parental cells and were randomized into different treatment groups.
Blinding	Oxygen consumption assay and histology assay were performed by the author who was blinded to the experimental groups. Other experiments were not blinded so that we could ensure each group had appropriate treatment or interventions.

Reporting for specific materials, systems and methods

We require information from authors about some types of materials, experimental systems and methods used in many studies. Here, indicate whether each material, system or method listed is relevant to your study. If you are not sure if a list item applies to your research, read the appropriate section before selecting a response.

Materials & experimental systems

n/a	Involved in the study	
☐	☒	Antibodies
☐	☒	Eukaryotic cell lines
☒	☐	Palaeontology and archaeology
☐	☒	Animals and other organisms
☒	☐	Clinical data
☒	☐	Dual use research of concern

Methods

n/a	Involved in the study	
☒	☐	ChIP-seq
☒	☐	Flow cytometry
☒	☐	MRI-based neuroimaging

Antibodies

Antibodies used	The following antibodies were used for western blot. They are listed as antibody name first, followed by supplier, catalog

number,clone name and dilution ratio:
(1) Rabbit polyclonal Flag antibody, Sigma, #F7425, N/A, 1:2000
(2) Rabbit polyclonal Adissp antibody, MyBioSourse, #MBS1493234, N/A, 1:200
(3) Rabbit monoclonal Adiponectin antibody, Cell Signaling Technology, #2789, C45B10, 1:500
(4) Rabbit polyclonal Ucp1 antibody, Sigma, #U6382, N/A, 1:1000
(5) Rabbit monoclonal Phospho-PKA Substrate antibody, Cell signaling Technology, #9624, 100G7E, 1:1000
(6) Rabbit polyclonal HSL antibody, Cell Signaling Technology, #4107, N/A, 1:1000
(7) Rabbit polyclonal Phospho-HSL (Ser660) Antibody, Cell Signaling Technology, #45804, N/A, 1:1000
(8) Mouse monoclonal Tubulin antibody, DSHB, #E7, E7, 1:5000
(9) Mouse monoclonal Actin antibody, Santa Cruz, sc-47778, C4, 1:2000
(10) Mouse anti-rabbit IgG-HRP, Santa Cruz, sc-2357, N/A, 1:2000
(11) Goat anti-Mouse IgG Antibody with (H+L) HRP conjugate, Sigma, AP308P, N/A, 1:2000

The following antibodies were used for immunofluorescence. They are listed as antibody name first, followed by supplier, catalog number, clone name and dilution ratio:
(1) Rabbit polyclonal Adissp antibody, MyBioSourse, #MBS1493234, N/A, 1:200
(2) Rabbit polyclonal Ucp1 antibody, Sigma, #U6382, N/A, 1:500
(3) Mouse monoclonal Actin antibody, Santa Cruz, sc-47778, C4, 1:200
(4) Goat anti-Rabbit IgG (H+L) Highly Cross-Adsorbed Secondary Antibody, Alexa Fluor™ 488, Thermo Fisher Scientific, A-11034, N/A, 1:1000
(5) Goat anti-Mouse IgG (H+L) Highly Cross-Adsorbed Secondary Antibody, Alexa Fluor™ 594, Thermo Fisher Scientific, A-11032, N/A, 1:1000
(6) Streptavidin with Alexa Fluor 488-Conjugate, Thermo Fisher Scientific, S32354, N/A, 1:500

Validation

The specificity of Adissp antibody (MyBioSourse, #MBS1493234) was evaluated using BAT tissue from WT and Adissp knockout mouse and adipocytes over-expression of Adissp.
Except for Adissp antibody, all antibodies were commercially available, and validated by manufacturers and/or citations. Manufacturer websites containing their validation data and/or citations, are listed below:
(1) Rabbit polyclonal Flag antibody (Sigma, #F7425): website (https://www.sigmaaldrich.com/US/en/product/sigma/f7425)
(2) Rabbit monoclonal Adiponectin antibody (Cell Signaling Technology, #2789): website (https://www.cellsignal.com/products/primary-antibodies/adiponectin-c45b10-rabbit-mab/2789) and citations (PMID: 34779857 (mouse), etc).
(3) Rabbit polyclonal Ucp1 antibody (Sigma, #U6382): website (https://www.sigmaaldrich.com/US/en/product/sigma/u6382?gclid=CjwKCAjw79iaBhAJEiwAPYwoCORv_qCbruuXp01RFMpF_755DU5RJDKo9SAoLLY79kfFvA3FYkpECxoCAPAQAvD_BwE&gclsrc=aw.ds) and citations (PMID: 22545021 (mouse), etc).
(4) Rabbit monoclonal Phospho-PKA Substrate antibody (Cell signaling Technology, #9624): website (https://www.cellsignal.com/products/primary-antibodies/phospho-pka-substrate-rrxs-t-100g7e-rabbit-mab/9624) and citations (PMID: 34016966 (mouse) and etc).
(5) Rabbit polyclonal HSL antibody (Cell Signaling Technology, #4107): website (https://www.cellsignal.com/products/primary-antibodies/hsl-antibody/4107) and citations (PMID: 35835749 (mouse) etc).
(6) Rabbit polyclonal Phospho-HSL (Ser660) Antibody (Cell Signaling Technology, #45804): website: (https://www.cellsignal.com/products/primary-antibodies/phospho-hsl-ser660-antibody/45804) and citations (PMID: 35835749(mouse) etc).
(7) Mouse monoclonal Tubulin antibody (DSHB, #E7): website (https://dshb.biology.uiowa.edu/E7_2) and citations (PMID: 24966384 (mouse) and etc).
(8) Mouse monoclonal Actin antibody (Santa Cruz, #sc-47778): website (https://www.scbt.com/p/beta-actin-antibody-c4?gclid=CjwKCAjw79iaBhAJEiwAPYwoCJsHx5kMnC335Q-lueSOd64hshHh35QA3TU0YSK8kC1OqAXAsLpUzBoChNQQAvD_BwE) and citations (PMID: 36257954 (mouse) etc).

Eukaryotic cell lines

Policy information about cell lines and Sex and Gender in Research

Cell line source(s)

HEK293 cell line used in this study was purchased from ATCC. Immortalized brown preadipocytes were generated by our lab previously (Pan et al. Cell, 2009). Primary progenitor cells used in this study were isolated from inguinal WAT of experimental housed mice.

Authentication

No authentication was used.

Mycoplasma contamination

They were not tested.

Commonly misidentified lines
(See ICLAC register)

No commonly misidentified cell line was used.

Animals and other research organisms

Policy information about studies involving animals; ARRIVE guidelines recommended for reporting animal research, and Sex and Gender in Research

Laboratory animals

Mice of both sexes aged 8-16 weeks were used for the experiments and stated in the figure legends. Littermate controls with same sex were used. All the mice were housed at 23 ± 1°C , 50% ± 20% humidity, 12 hr light/dark cycles and had free access to food and water. C57BL/6J wild type mice (Stock No. 000664), Flp mice (Stock No. 012930) were obtained from the Jackson Laboratory. Adissp transgenic and knockout mice were generated by core facility of UMASS Medical School. Adiponectin-cre mice were published

	(Eguchi et al. 2011) and available in the Jackson Laboratory. Ucp1 knockout mice were get from David Guertin lab in UMASS Chan Medical School.
Wild animals	This study did not involve wild animals.
Reporting on sex	Inguinal WAT browning, body temperature, and blood glucose were analyzed in both male and female mice, and similar results were obtained. Based on these observations, we think there is no difference in phenotypes between male and female mice, and therefore some other experiments, such as HFD experiments, were done with male mice, which is a common practice in the field.
Field-collected samples	This study did not involve samples collected from the field.
Ethics oversight	All animal studies were performed according to procedures approved by the UMASS Chan Medical School's Institutional Animal Care and Use Committee (IACUC), Protocol number is 202000027.

Note that full information on the approval of the study protocol must also be provided in the manuscript.
